# Criterion for Assessing Accumulated Neurotoxicity of Alpha‐Synuclein Oligomers in Parkinson's Disease

**DOI:** 10.1002/cnm.70027

**Published:** 2025-04-28

**Authors:** Andrey V. Kuznetsov

**Affiliations:** ^1^ Department of Mechanical and Aerospace Engineering North Carolina State University Raleigh North Carolina USA

**Keywords:** α‐Synucleinopathy, Finke‐Watzky, Lewy body, mathematical modeling, neuron

## Abstract

The paper introduces a parameter called “accumulated neurotoxicity” of α‐syn oligomers, which measures the cumulative damage these toxic species inflict on neurons over time, given the years it typically takes for such damage to manifest. A threshold value for accumulated neurotoxicity is estimated, beyond which neuron death is likely. Numerical results suggest that rapid deposition of α‐syn oligomers into fibrils minimizes neurotoxicity, indicating that the formation of Lewy bodies might play a neuroprotective role. Strategies such as reducing α‐syn monomer production or enhancing degradation can decrease accumulated neurotoxicity. In contrast, slower degradation (reflected by longer half‐lives of monomers and free aggregates) increases neurotoxicity, supporting the idea that impaired protein degradation may contribute to Parkinson's disease progression. Accumulated neurotoxicity is highly sensitive to the half‐deposition time of free α‐syn aggregates into fibrils, exhibiting a sharp increase as it transitions from negligible to elevated levels, indicative of neural damage.

## Graphical abstract

The study introduces a new parameter, “accumulated neurotoxicity,” to evaluate the long‐term damage caused by α‐synuclein (α‐syn) oligomers in Parkinson's disease. The results suggest that rapid deposition of α‐syn oligomers into fibrils reduces toxicity, indicating that Lewy bodies may play a neuroprotective role. Impaired protein degradation, reflected in prolonged half‐lives of α‐syn monomers and aggregates, increases toxicity. Therapeutic strategies, such as enhancing α‐syn degradation or reducing its production, may help reduce toxicity and slow disease progression.

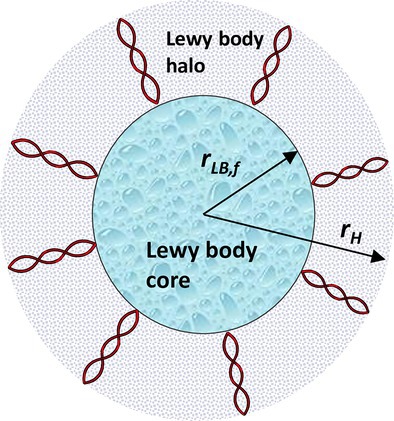

**Abbreviations**
F‐W: Finke‐Watzky
LB: Lewy body
PD: Parkinson's disease
α‐syn: α‐synuclein

## 1 Introduction

Parkinson's disease (PD) is a neurodegenerative condition marked by the degeneration of dopaminergic neurons in the substantia nigra, accompanied by the formation of Lewy bodies (LBs), which are abnormal protein aggregates found within affected neurons [1, 2, 3, 4, 5].

Alpha‐synuclein (α‐syn), a key protein implicated in PD, undergoes misfolding and aggregation, resulting in the formation of oligomers that contribute to neuronal damage and neurotoxicity [6]. Recent research suggests that LBs, traditionally viewed as toxic deposits of misfolded and aggregated α‐syn within neurons, may be neuroprotective and sequester toxic α‐syn oligomers [7]. This emerging perspective challenges the conventional view of LBs as purely pathological. Consequently, the relationship between LB formation and the accumulation of α‐syn oligomers in the cytosol has become a critical area of investigation. Understanding the role α‐syn aggregates play in neurotoxicity and how their sequestration into LBs impacts disease progression is essential for developing therapeutic strategies targeting alpha‐synucleinopathies [8].

Classical LBs [9] were traditionally understood to consist primarily of α‐syn fibrils. This hypothesis was supported by extensive experimental evidence [10, 11, 12]. However, recent findings [13] reveal that LBs also contain lipid membrane fragments and altered organelles, challenging the previous notion that α‐syn fibrils are the main components of LBs. This discovery has sparked ongoing debates about the role of α‐syn fibrils within LBs and LB composition [12, 14], suggesting that LBs may represent more complex aggregates of various intracellular materials. Classical brainstem‐type LBs are characterized by a dense central core surrounded by a halo (an outer layer) of radiating filaments [11, 14]. Both the core and the halo exhibit a spherical structure. It is likely that LBs initially develop from pale bodies, which are granular entities lacking a halo [14]. Over time, these pale bodies can evolve into the classical LBs with a surrounding halo [15].

**FIGURE 1:**
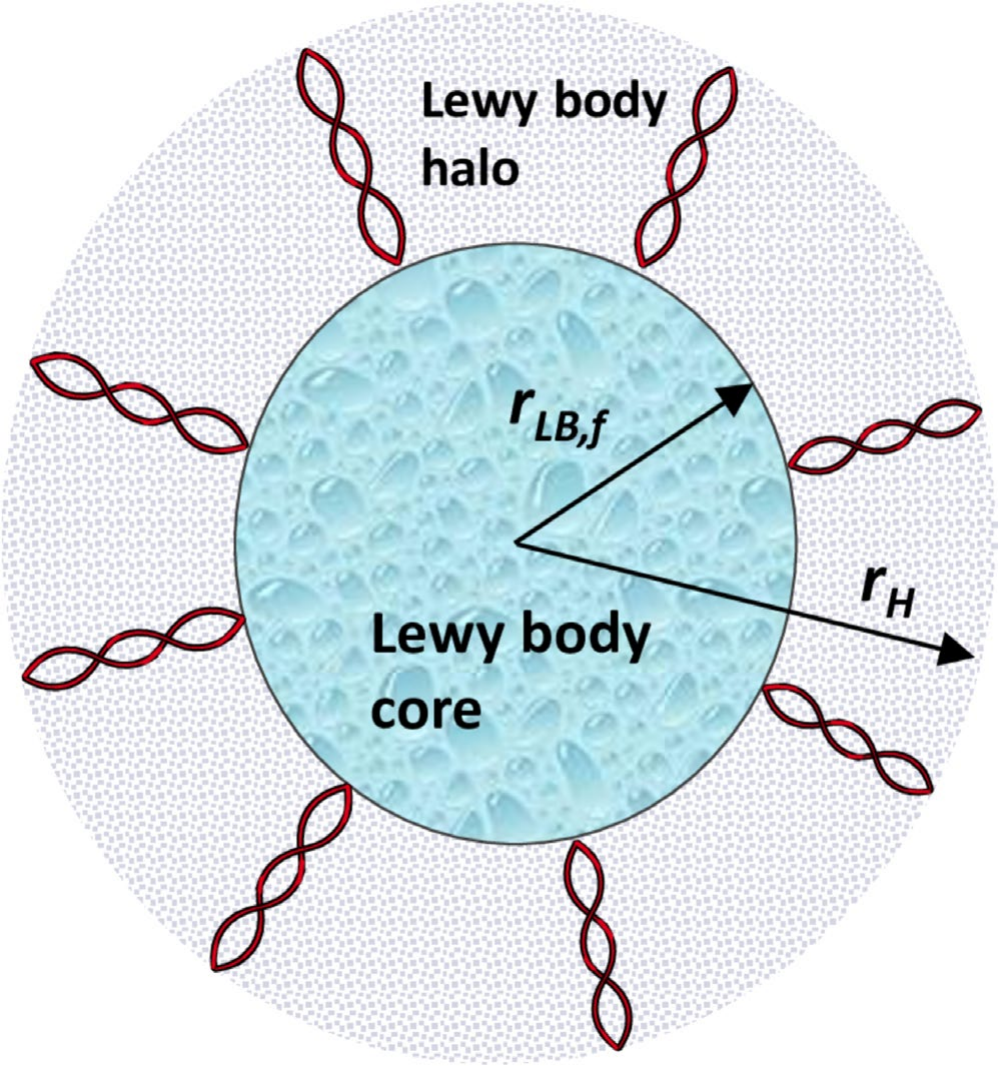
A diagram illustrating an LB, composed of two distinct regions: A central core filled with dense granular material, and a surrounding halo characterized by radiating filaments. This depiction is based on electron micrographs shown in Figure 1 of reference [17] and Figure 3 of reference [14]. Figure generated with the aid of servier medical art, licensed under a creative commons attribution 3.0 generic license. http://Smart.servier.com.

To address these complexities, in reference [16], a two‐stage model of LB formation was introduced. Drawing from recent experimental findings showing that classical LBs feature a dense core surrounded by a halo of radiating filaments [12, 13], the model suggests that the core of the LB develops from the aggregation of lipid membrane fragments and altered organelles. In the second stage, the surface of this core triggers the growth of α‐syn fibrils, which incorporate α‐syn monomers from the cytosol (Figure 1). This model not only explains the structural organization of LBs but also shows how fibril formation restricts further core growth.

Developing a criterion to assess brain tissue damage at the cellular scale is important for optimizing treatments for neurodegenerative diseases [18, 19, 20]. Recent research indicates that soluble α‐syn oligomers, which are intermediates in the formation of α‐syn fibrils, may be more toxic than LBs [21]. The forming of these oligomeric species is a crucial step in the α‐syn cascade hypothesis [22]. One potential mechanism underlying the neurotoxicity of α‐syn oligomers involves their ability to disrupt membrane integrity [23]. This study introduces a criterion for evaluating accumulated neurotoxicity. Accumulated neurotoxicity serves as a unified metric for quantifying neurotoxicity, enabling a clearer understanding and comparison of neurotoxicity profiles across different cases.

Additionally, this study conducts a numerical analysis of α‐syn aggregation, with a particular emphasis on the accumulation of neurotoxicity over time as α‐syn oligomers either persist in the cytosol or become incorporated into LBs. Building on previous research on LB growth dynamics [16, 24] and recent work on the concept of accumulated neurotoxicity of Aβ and tau oligomers [25, 26], this investigation seeks to identify the key factors driving neurotoxicity in PD. It specifically examines how the sensitivity of accumulated neurotoxicity responds to the deposition rates of α‐syn oligomers into LBs, analyzing their impact on the expansion of the LB halo and overall neuronal health. It also examines how the half‐lives of α‐syn monomers and aggregates impact the progression of PD, highlighting the connection between impaired protein degradation pathways and disease development.

The results suggest that rapid deposition of α‐syn oligomers into LBs may significantly reduce accumulated neurotoxicity, supporting the notion that LBs could mitigate neurotoxic effects. In contrast, slow deposition allows oligomers to remain in the cytosol, where they continue to catalyze their own production and exacerbate neurotoxicity. These insights provide a framework for assessing the role of α‐syn oligomers in PD and offer potential avenues for therapeutic intervention aimed at reducing their toxic effects.

## 2 Materials and Models

### 2.1 Equations Governing Lipid Membrane Fragment Aggregation in LB's Core

A minimal two‐step Finke‐Watzky (F‐W) model was applied to simulate the aggregation of lipid fragments in the soma, based on approaches outlined in references [27, 28]. This model captures polymer aggregation through two pseudoelementary processes: continuous nucleation and autocatalytic surface growth [29]. The F‐W model has previously been utilized to analyze the aggregation behavior of neurological proteins [27]. The two pseudoelementary steps in the F‐W model are described by the following equations:

(1) $$A\overset{k_{1}}{\to }B$$

(2) $$A+B\overset{k_{2}}{\to }2B$$

The F‐W model is typically used to simulate the transformation of a given initial concentration of monomers (*A*) into misfolded or autocatalytically active aggregates (*B*) [28]. While analytical solutions for such processes are well documented (e.g., reference [30]), this study adapts the F‐W model to a different context by simulating the scenario where monomers are continuously produced. Recent findings [13, 14] indicate that the core of LBs mainly consists of lipid membrane fragments and damaged organelles, referred to here simply as membrane fragments.

The model uses time, *t*, as the sole independent variable. The dependent variables are detailed in Table S1 of the Supporting Informations, and Table S2 summarizes the parameters used in the model, which are estimated from data reported in references [17, 31, 32, 33, 34, 35, 36, 37, 38, 39].

The production rate of membrane fragments in the soma, $q_{\mathrm{FR}}$, is assumed to be constant. By considering the soma as the control volume and applying the principle of conservation of membrane fragments within this control volume, the following equation is derived:

(3) $$\frac{d\left[A_{\mathrm{FR}}\right]}{\mathrm{dt}}=\frac{q_{\mathrm{FR}}}{V_{\text{soma}}}a_{21}-k_{1,\mathrm{FR}}\left[A_{\mathrm{FR}}\right]-k_{2,\mathrm{FR}}\left[A_{\mathrm{FR}}\right]\left[B_{\mathrm{FR}}\right]-\left[A_{\mathrm{FR}}\right]\frac{\ln\left(2\right)}{T_{1/2,A,\mathrm{FR}}}$$

In this equation, $\left[A_{\mathrm{FR}}\right]$ represents the concentration of membrane fragments, $\left[B_{\mathrm{FR}}\right]$ denotes the concentration of free lipid membrane aggregates, which can form the LB core, and $a_{21}$ is the conversion factor from $\frac{\mathrm{mol}\;}{\mu m^{3}\;}$ to μM ($10^{21}$ $\frac{\mu M\;\mu m^{3}}{\mathrm{mol}\;}$). The parameters $k_{1,\mathrm{FR}}$ and $k_{2,\mathrm{FR}}$ are rate constants for the nucleation and autocatalytic growth of lipid membrane fragment aggregates, respectively. The quantity $T_{1/2,A,\mathrm{FR}}$ is the half‐life of lipid membrane fragments, and $V_{\text{soma}}$ is the volume of the soma.

The right‐hand side of Equation (3) includes several terms. The first term represents the production rate of membrane fragments. The second term models the conversion of these fragments into aggregates through nucleation, while the third term accounts for the conversion through autocatalytic growth. The final term models the degradation of membrane fragments.

By stating the conservation of membrane aggregates in the soma, the following equation is derived:

(4) $$\frac{d\left[B_{\mathrm{FR}}\right]}{\mathrm{dt}}=k_{1,\mathrm{FR}}\left[A_{\mathrm{FR}}\right]+k_{2,\mathrm{FR}}\left[A_{\mathrm{FR}}\right]\left[B_{\mathrm{FR}}\right]-\left[B_{\mathrm{FR}}\right]\frac{\ln\left(2\right)}{T_{1/2,B,\mathrm{FR}}}-\left[B_{\mathrm{FR}}\right]\frac{\ln\left(2\right)}{\theta_{1/2,B,\mathrm{FR}}}$$

where $T_{1/2,B,\mathrm{FR}}$ represents the half‐life of free aggregated lipid membrane fragments (those not yet deposited into the LB core), and $\theta_{1/2,B,\mathrm{FR}}$ denotes the half‐deposition time of free lipid membrane aggregates into the LB core. On the right‐hand side, the first term captures the nucleation‐driven rate of free aggregate production from monomers, while the second term describes the rate of free aggregate production through autocatalytic growth. These terms mirror the corresponding ones in Equation (3) but with opposite signs, as the F‐W model assumes that aggregate production equals monomer consumption. The third term models the degradation of lipid membrane aggregates, and the fourth term represents their deposition into the LB core.

The process of LB core formation from adhesive lipid fragment aggregates is modeled by an approach akin to colloidal suspension coagulation [40]. In this framework, free lipid fragment aggregates (*B*) are assumed to deposit into the LB core, with the concentration of these deposited aggregates represented by $\left[D_{\mathrm{FR}}\right]$:

(5) $$\frac{d\left[D_{\mathrm{FR}}\right]}{\mathrm{dt}}=\left[B_{\mathrm{FR}}\right]\frac{\ln\left(2\right)}{\theta_{1/2,B,\mathrm{FR}}}-\left[D_{\mathrm{FR}}\right]\frac{\ln\left(2\right)}{T_{1/2,D,\mathrm{FR}}}$$

where $\left[D_{\mathrm{FR}}\right]$ represents the concentration of lipid membrane aggregates deposited into the LB core, while $T_{1/2,D,\mathrm{FR}}$ is the half‐life of these deposited aggregates.

In Equation (5), the first term on the right‐hand side mirrors the fourth term in Equation (4), but with the opposite sign, indicating the deposition process. The second term accounts for the degradation of lipid fragment aggregates within the LB core, reflecting their possible finite half‐life. This term anticipates potential future therapies aimed at LB clearance, possibly through autophagy, as discussed in reference [41].

Equations ((3), (4), (5)) are solved under the following initial conditions:

(6) $$\mathrm{At}\;t=0:\;\left[A_{\mathrm{FR}}\right]={\left[A_{\mathrm{FR}}\right]}_{0}\;\left(a\right),\;\left[B_{\mathrm{FR}}\right]=0\;\left(b\right),\;\left[D_{\mathrm{FR}}\right]=0\;\left(c\right)$$

where ${\left[A_{\mathrm{FR}}\right]}_{0}$ represents the initial concentration of lipid membrane fragments within the soma at the start of the process.

### 2.2 Analytical Solutions for the Limiting Case of Slow Deposition of Free Lipid Membrane Aggregates Into the LB Core, $\theta_{1/2,B,\mathrm{FR}}\to \infty$ (and also $T_{1/2,A,\mathrm{FR}}\to \infty$, $T_{1/2,B,\mathrm{FR}}\to \infty$, and $T_{1/2,D,\mathrm{FR}}\to \infty$)

If the deposition rate of free lipid membrane aggregates into the LB core is slow and the half‐lives of monomers, free aggregates, and deposited aggregates are infinitely long, Equations ((3), (4), (5)) simplify to the following reduced forms:

(7) $$\frac{d\left[A_{\mathrm{FR}}\right]}{\mathrm{dt}}=\frac{q_{\mathrm{FR}}}{V_{\text{soma}}}a_{21}-k_{1,\mathrm{FR}}\left[A_{\mathrm{FR}}\right]-k_{2,\mathrm{FR}}\left[A_{\mathrm{FR}}\right]\left[B_{\mathrm{FR}}\right]$$

(8) $$\frac{d\left[B_{\mathrm{FR}}\right]}{\mathrm{dt}}=k_{1,\mathrm{FR}}\left[A_{\mathrm{FR}}\right]+k_{2,\mathrm{FR}}\left[A_{\mathrm{FR}}\right]\left[B_{\mathrm{FR}}\right]$$

(9) $$\frac{d\left[D_{\mathrm{FR}}\right]}{\mathrm{dt}}=0$$

To streamline the analysis, it is assumed that the initial concentrations of lipid membrane fragments are zero—that is,

(10) $$\mathrm{At}\;t=0:\;\left[A_{\mathrm{FR}}\right]=0\;\left(a\right),\;\left[B_{\mathrm{FR}}\right]=0\;\left(b\right),\;\left[D_{\mathrm{FR}}\right]=0\;\left(c\right)$$

The numerical solution of Equations ((7), (8), (9), (10)) shows that $\left[A_{\mathrm{FR}}\right]\to 0$ as t→∞. Based on this observation, an approximate solution for Equations (7) and (8), valid for large *t*, was derived in reference [16] as follows:

(11) $$\left[B_{\mathrm{FR}}\right]=\frac{q_{\mathrm{FR}}}{V_{\text{soma}}}a_{21}t$$

(12) $$\left[A_{\mathrm{FR}}\right]=\frac{\frac{q_{\mathrm{FR}}}{V_{\text{soma}}}a_{21}\frac{1}{k_{1,\mathrm{FR}}}}{1+\frac{q_{\mathrm{FR}}}{V_{\text{soma}}}a_{21}\frac{k_{2,\mathrm{FR}}}{k_{1,\mathrm{FR}}}t}$$

Also, from Equations (9) and (10c), it follows that:

(13) $$\left[D_{\mathrm{FR}}\right]=0$$

### 2.3 A Model for Simulating LB Core Growth

The growth of the LB core (Figure 1) is determined by calculating the total number of membrane fragments incorporated into the core, $N_{\mathrm{LB}}$, over time *t*. This is done using the following approach, adapted from reference [42]:

(14) $$N_{\mathrm{LB}}\left(t\right)=\frac{\left[D_{\mathrm{FR}}\right]\left(t\right)}{a_{21}}V_{\text{soma}}N_{A}$$

where $N_{A}$ is Avogadro's number.

Alternatively, $N_{\mathrm{LB}}\left(t\right)$ can be determined using the following equation, as outlined in reference [42]:

(15) $$N_{\mathrm{LB}}\left(t\right)=\frac{V_{\mathrm{LB}}\left(t\right)\rho_{\mathrm{LB}}}{{\mathrm{MW}}_{\mathrm{FR}}}N_{A}$$

where ${\mathrm{MW}}_{\mathrm{FR}}$ represents the average molecular weight of a lipid membrane fragment, calculated as the sum of the atomic weights of all atoms within the fragment, $V_{\mathrm{LB}}\left(t\right)$ is the volume of the LB core at time *t*, and $\rho_{\mathrm{LB}}$ denotes the density of the LB, assumed to be the same for both the core and halo regions.

Equating the right‐hand sides of Equations (14) and (15) and solving for the LB core volume yields:

(16) $$V_{\mathrm{LB}}\left(t\right)=\frac{\left[D_{\mathrm{FR}}\right]\left(t\right)}{a_{21}}V_{\text{soma}}\frac{{\mathrm{MW}}_{\mathrm{FR}}}{\rho_{\mathrm{LB}}}$$

Assuming the LB core maintains a spherical geometry, the volume of the core can be expressed as:

(17) $$V_{\mathrm{LB}}\left(t\right)=\frac{4}{3}\pi r_{\mathrm{LB}}^{3}\left(t\right)$$

where $r_{\mathrm{LB}}$ represents the radius of the LB core.

Solving Equations (16) and (17) for the core radius yields:

(18) $$r_{\mathrm{LB}}={\left(\frac{3}{4\pi }\frac{\left[D_{\mathrm{FR}}\right]}{a_{21}}V_{\text{soma}}\frac{{\mathrm{MW}}_{\mathrm{FR}}}{\rho_{\mathrm{LB}}}\right)}^{1/3}$$

By substituting Equation (13) into Equation (18), the following result is obtained:

(19) $$r_{\mathrm{LB}}=0$$

However, if it is assumed that all free membrane fragment aggregates eventually deposit into the core of the LB (note that this differs from the assumption that $\theta_{1/2,B,\mathrm{FR}}\to \infty$, and introduces an additional assumption), the term $\left[D_{\mathrm{FR}}\right]$ in Equation (18) can be substituted by $\left[B_{\mathrm{FR}}\right]$ from Equation (11), resulting in:

(20) $$r_{\mathrm{LB}}={\left(\frac{3}{4\pi }q_{\mathrm{FR}}\frac{{\mathrm{MW}}_{\mathrm{FR}}}{\rho_{\mathrm{LB}}}\right)}^{1/3}t^{1/3}$$

The LB core grows until time $t_{\mathrm{LB},f}$, at which point $r_{\mathrm{LB}}$ reaches $r_{\mathrm{LB},f}$. Solving Equation (20) for $q_{\mathrm{FR}}$ under these conditions gives:

(21) $$q_{\mathrm{FR}}=\frac{4\pi }{3}\frac{\rho_{\mathrm{LB}}}{{\mathrm{MW}}_{\mathrm{FR}}}\frac{r_{\mathrm{LB},f}^{3}}{t_{\mathrm{LB},f}}$$

Equation (21) is essential for estimating model parameters, as it allows for the calculation of $q_{\mathrm{FR}}$ when $r_{\mathrm{LB},f}$ and $t_{\mathrm{LB},f}$ are known. This equation estimates $q_{\mathrm{FR}}$ to be $1.57\times 10^{-28}$ mol s^−1^, as listed in Table S2.

### 2.4 Equations for Simulating the Aggregation of α‐Syn Monomers in the LB Halo

A constant production rate of α‐syn monomers in the soma, $q_{\mathrm{AS}}$, is assumed. The F‐W model [27], detailed in Equations ((3), (4), (5)), is employed to simulate the formation of α‐syn fibrils in the soma. Stating the conservation of α‐syn monomers in the soma gives:

(22) $$\frac{d\left[A_{\mathrm{AS}}\right]}{\mathrm{dt}}=\frac{q_{\mathrm{AS}}}{V_{\text{soma}}}a_{21}-k_{1,\mathrm{AS}}\left[A_{\mathrm{AS}}\right]-k_{2,\mathrm{AS}}\left[A_{\mathrm{AS}}\right]\left[B_{\mathrm{AS}}\right]-\left[A_{\mathrm{AS}}\right]\frac{\ln\left(2\right)}{T_{1/2,A,\mathrm{AS}}}$$

where $\left[A_{\mathrm{AS}}\right]$ represents the concentration of α‐syn monomers in the soma, $\left[B_{\mathrm{AS}}\right]$ denotes the concentration of free α‐syn aggregates in the soma, $k_{1,\mathrm{AS}}$ is the rate constant for the nucleation of free α‐syn aggregates, $k_{2,\mathrm{AS}}$ is the rate constant for the autocatalytic growth of these aggregates, and $T_{1/2,A,\mathrm{AS}}$ is the half‐life of α‐syn monomers. Hereafter the words free α‐syn aggregates and α‐syn oligomers will be used as synonyms.

In Equation (22), the first term on the right‐hand side accounts for the production rate of α‐syn monomers. The second and third terms describe the transformation of α‐syn monomers into free aggregates via nucleation and autocatalytic growth. The fourth term reflects the decay of α‐syn monomers due to their finite half‐life.

Applying the conservation to free α‐syn aggregates yields:

(23) $$\frac{d\left[B_{\mathrm{AS}}\right]}{\mathrm{dt}}=k_{1,\mathrm{AS}}\left[A_{\mathrm{AS}}\right]+k_{2,\mathrm{AS}}\left[A_{\mathrm{AS}}\right]\left[B_{\mathrm{AS}}\right]-\left[B_{\mathrm{AS}}\right]\frac{\ln\left(2\right)}{T_{1/2,B,\mathrm{AS}}}-\left[B_{\mathrm{AS}}\right]\frac{\ln\left(2\right)}{\theta_{1/2,B,\mathrm{AS}}}$$

where $\theta_{1/2,B,\mathrm{AS}}$ represents the half‐deposition time of free α‐syn aggregates into fibrils.

In Equation (23), the first two terms represent the increase in free α‐syn aggregates due to nucleation and autocatalytic growth, while the third term models their degradation based on their half‐life. The fourth term in Equation (23) represents the reduction in free α‐syn aggregates as they are deposited into fibrils. Equations (22) and (23) are analogous to Equations (3) and (4), respectively.

The process of α‐syn fibril formation in the LB halo from free α‐syn aggregates is modeled similarly to colloidal suspension coagulation [40]. In this approach, free α‐syn aggregates (*B*) are assumed to deposit into fibrils that constitute the LB halo, with their concentration denoted by $\left[D_{\mathrm{AS}}\right]$. This results in an equation similar to Equation (5):

(24) $$\frac{d\left[D_{\mathrm{AS}}\right]}{\mathrm{dt}}=\left[B_{\mathrm{AS}}\right]\frac{\ln\left(2\right)}{\theta_{1/2,B,\mathrm{AS}}}-\left[D_{\mathrm{AS}}\right]\frac{\ln\left(2\right)}{T_{1/2,D,\mathrm{AS}}}$$

where $\left[D_{\mathrm{AS}}\right]$ denotes the concentration of α‐syn aggregates deposited into fibrils in the LB halo, and $T_{1/2,D,\mathrm{AS}}$ represents the half‐life of these deposited aggregates. In Equation (24), the first term on the right‐hand side corresponds to the fourth term in Equation (23) but with the opposite sign, reflecting the deposition of free aggregates. The second term models the degradation of α‐syn aggregates within the fibrils if their half‐life is finite. This term also accounts for potential therapeutic interventions, such as autophagy‐based therapies [41], which may aim to clear LBs.

Equations ((22), (23), (24)) are solved under the following initial conditions:

(25) $$\mathrm{At}\;t=t_{\mathrm{LB},f}:\;\left[A_{\mathrm{AS}}\right]={\left[A_{\mathrm{AS}}\right]}_{0}\;\left(a\right),\;\left[B_{\mathrm{AS}}\right]=0\;\left(b\right),\;\left[D_{AS}\right]=0\;\left(c\right)$$

where ${\left[A_{\mathrm{AS}}\right]}_{0}$ represents the initial concentration of α‐syn monomers in the soma.

### 2.5 Analytical Solutions for the Limiting Case of Slow Deposition of Free α‐Syn Aggregates Into Fibrils, $\theta_{1/2,B,\mathrm{AS}}\to \infty$ (and also $T_{1/2,A,\mathrm{AS}}\to \infty$, $T_{1/2,B,\mathrm{AS}}\to \infty$, and $T_{1/2,D,\mathrm{AS}}\to \infty$)

In the case where the deposition rate of free α‐syn aggregates into fibrils is slow and the half‐lives of α‐syn monomers, free aggregates, and deposited aggregates are assumed to be infinitely large, the governing Equations ((22), (23), (24), (25)) simplify significantly, yielding the following expressions:

(26) $$\frac{d\left[A_{\mathrm{AS}}\right]}{\mathrm{dt}}=\frac{q_{\mathrm{AS}}}{V_{\text{soma}}}a_{21}-k_{1,\mathrm{AS}}\left[A_{\mathrm{AS}}\right]-k_{2,\mathrm{AS}}\left[A_{\mathrm{AS}}\right]\left[B_{\mathrm{AS}}\right]$$

(27) $$\frac{d\left[B_{\mathrm{AS}}\right]}{\mathrm{dt}}=k_{1,\mathrm{AS}}\left[A_{\mathrm{AS}}\right]+k_{2,\mathrm{AS}}\left[A_{\mathrm{AS}}\right]\left[B_{\mathrm{AS}}\right]$$

(28) $$\frac{d\left[D_{\mathrm{AS}}\right]}{\mathrm{dt}}=0$$

To simplify the analysis, the initial concentration of α‐syn monomers in the soma is set to zero, leading to the following initial conditions:

(29) $$\mathrm{At}\;t=t_{\mathrm{LB},f}:\;\left[A_{\mathrm{AS}}\right]=0\;\left(a\right),\;\left[B_{\mathrm{AS}}\right]=0\;\left(b\right),\;\left[D_{AS}\right]=0\;\left(c\right)$$

Numerical analysis of Equations ((26), (27), (28), (29)) indicates that as time increases, $\left[A_{\mathrm{AS}}\right]\to 0$ as t→∞. Based on this observation, an approximate solution for Equations (26) and (27), applicable for large values of *t*, was derived in reference [16] as follows:

(30) $$\left[B_{\mathrm{AS}}\right]=\frac{q_{\mathrm{AS}}}{V_{\text{soma}}}a_{21}\left(t-t_{\mathrm{LB},f}\right)$$

(31) $$\left[A_{\mathrm{AS}}\right]=\frac{\frac{q_{\mathrm{AS}}}{V_{\text{soma}}}a_{21}\frac{1}{k_{1,\mathrm{AS}}}}{1+\frac{q_{\mathrm{AS}}}{V_{\text{soma}}}a_{21}\frac{k_{2,\mathrm{AS}}}{k_{1,\mathrm{AS}}}\left(t-t_{\mathrm{LB},f}\right)}$$

Also, from Equations (28) and (29c) it follows that:

(32) $$\left[D_{AS}\right]=0$$

### 2.6 Method for Simulating the Growth of the LB Halo

To model the growth of the halo surrounding the LB core (Figure 1), the total number of α‐syn monomers incorporated into the halo at time *t*, denoted as $N_{\mathrm{AS}}$, is calculated using the following approach [42]:

(33) $$N_{\mathrm{AS}}\left(t\right)=\frac{\left[D_{\mathrm{AS}}\right]\left(t\right)}{a_{21}}V_{\text{soma}}N_{A}$$

Alternatively, $N_{\mathrm{AS}}\left(t\right)$ can be determined using the equation provided in reference [42]:

(34) $$N_{\mathrm{AS}}\left(t\right)=\frac{V_{H}\left(t\right)\rho_{\mathrm{LB}}}{{\mathrm{MW}}_{\mathrm{AS}}}N_{A}$$

where ${\mathrm{MW}}_{\mathrm{AS}}$ denotes the molecular weight of an α‐syn monomer and $V_{H}$ represents the volume of the LB halo at time *t*.

By equating the right‐hand sides of Equations (33) and (34) and solving for the LB volume, the following result is obtained:

(35) $$V_{H}\left(t\right)=\frac{\left[D_{\mathrm{AS}}\right]\left(t\right)}{a_{21}}V_{\text{soma}}\frac{{\mathrm{MW}}_{\mathrm{AS}}}{\rho_{\mathrm{LB}}}$$

Assuming the LB halo is shaped like a spherical shell,

(36) $$V_{H}\left(t\right)=\frac{4}{3}\pi \left(r_{H}^{3}-r_{\mathrm{LB},f}^{3}\right)$$

Solving Equations (35) and (36) for the halo radius $r_{H}$ yields:

(37) $$r_{H}={\left(r_{\mathrm{LB},f}^{3}+\frac{3}{4\pi }\frac{\left[D_{\mathrm{AS}}\right]}{a_{21}}V_{\text{soma}}\frac{{\mathrm{MW}}_{\mathrm{AS}}}{\rho_{\mathrm{LB}}}\right)}^{1/3}$$

Assuming that free α‐syn aggregates eventually deposit into the LB halo (which differs from the assumption that $\theta_{1/2,B,\mathrm{AS}}\to \infty$, and introduces an additional assumption), $\left[D_{\mathrm{AS}}\right]$ in Equation (37) can be replaced by $\left[B_{\mathrm{AS}}\right]$, as defined by Equation (30). Doing so gives:

(38) $$r_{H}={\left(r_{\mathrm{LB},f}^{3}+\frac{3}{4\pi }q_{\mathrm{AS}}\frac{{\mathrm{MW}}_{\mathrm{AS}}}{\rho_{\mathrm{LB}}}\left(t-t_{\mathrm{LB},f}\right)\right)}^{1/3}$$

The LB halo will keep growing until it reaches its maximum radius $r_{H,f}$, at which point *t* equals $t_{\mathrm{LB},f}+t_{H,f}$. To find $q_{\mathrm{AS}}$ Equation (38) was solved for these values of r and t, resulting in:

(39) $$q_{\mathrm{AS}}=\frac{4\pi }{3}\frac{\rho_{\mathrm{LB}}}{{\mathrm{MW}}_{\mathrm{AS}}}\frac{r_{H,f}^{3}-r_{\mathrm{LB},f}^{3}}{t_{H,f}-t_{\mathrm{LB},f}}$$

Equation (39) is crucial for estimating model parameters because it allows for the calculation of $q_{\mathrm{AS}}$ when the values of $r_{\mathrm{LB},f}$, $r_{H,f}$, and $t_{H,f}$ are known. Using Equation (39), $q_{\mathrm{AS}}$ was estimated to be $1.47\times 10^{-21}$ mol s^−1^, as reported in Table S2.

### 2.7 Criterion for Assessing Accumulated Neurotoxicity of α‐Syn Oligomers

This paper proposes a parameter to characterize the accumulated neurotoxicity of α‐syn oligomers. Accumulated neurotoxicity is represented by the symbol Ξ and mathematically defined as:

(40) $$\Xi \left(t\right)=\int_{t_{\mathrm{LB},f}}^{t}\left[B_{\mathrm{AS}}\right]\left(\hat{t}\right)d\hat{t}$$

Note that for $t<t_{\mathrm{LB},f}$, neurotoxicity is zero. The dimensionless form of Equation (40) is given by Equation (S1) in Supporting Information.

Using the approximate Equation (30) in Equation (40), it follows that for $t>t_{\mathrm{LB},f}$,

(41) $$\Xi \left(t\right)=\frac{a_{21}q_{\mathrm{AS}}}{V_{\text{soma}}}\frac{{\left(t-t_{\mathrm{LB},f}\right)}^{2}}{2}$$

The quadratic relationship between neurotoxicity and time implies that early in the growth of the halo, the accumulated neurotoxicity increases gradually, but over time, the rate of increase becomes significantly faster. Equation (41) is only valid as $\theta_{1/2,B,\mathrm{AS}}\to \infty$, $T_{1/2,A,\mathrm{AS}}\to \infty$, $T_{1/2,B,\mathrm{AS}}\to \infty$, and $T_{1/2,D,\mathrm{AS}}\to \infty$.

In the case where $\theta_{1/2,B,\mathrm{AS}}\to 0$, Ξ also approaches zero. The neurotoxicity of α‐syn oligomers (considered equivalent to free aggregates in the model) tends to zero because the aggregates immediately deposit into fibrils, bypassing the stage in which they remain in the cytosol as free aggregates.

### 2.8 Sensitivity of Accumulated Neurotoxicity of α‐Syn Oligomers to Model Parameters

This study aimed to assess how the accumulated neurotoxicity of α‐syn oligomers varies with different model parameters. This analysis involved calculating the local sensitivity coefficients, which are the first‐order partial derivatives of accumulated neurotoxicity with respect to parameters such as the half‐deposition time of free α‐syn aggregates into fibrils, $\theta_{1/2,B,\mathrm{AS}}$. The methodology used for this analysis was based on the approaches outlined in references [43, 44, 45, 46]. Specifically, the sensitivity coefficient of Ξ to a parameter $\theta_{1/2,B,\mathrm{AS}}$ was determined by:

(42) $$\frac{\partial \;\Xi }{\partial \theta_{1/2,B,\mathrm{AS}}}\approx {\left.\frac{\;\Xi \left(\;\theta_{1/2,B,\mathrm{AS}}+\Delta \theta_{1/2,B,\mathrm{AS}}\right)-\Xi \left(\theta_{1/2,B,\mathrm{AS}}\right)}{\Delta \theta_{1/2,B,\mathrm{AS}}}\right|}_{\text{other parmeters kept constant}}$$

where $\Delta \theta_{1/2,B,\mathrm{AS}}=10^{-3}\theta_{1/2,B,\mathrm{AS}}$ represents the step size. To ensure the sensitivity coefficients were independent of the chosen step size, calculations were performed using various step sizes.

The non‐dimensional relative sensitivity coefficients were calculated using the method outlined in references [44, 47], for example,

(43) $$S_{\theta_{1/2,B,\mathrm{AS}}}^{\Xi }=\frac{\theta_{1/2,B,\mathrm{AS}}}{\;\Xi }\frac{\partial \Xi }{\partial \theta_{1/2,B,\mathrm{AS}}}$$

By utilizing Equation (41), the result can be expressed as follows:

(44) $$S_{\theta_{1/2,B,\mathrm{AS}}}^{\Xi }=0$$

Additionally, utilizing Equation (41), the sensitivity of Ξ to various parameters is calculated as follows:

(45) $$S_{k_{1}}^{\Xi }=0$$

(46) $$S_{k_{2}}^{\Xi }=0$$

(47) $$S_{q_{\mathrm{AS}}}^{\Xi }=1$$

(48) $$S_{t_{H,f}}^{\Xi }=\frac{2t_{H,f}}{t_{H,f}-t_{\mathrm{LB},f}}$$

(49) $$S_{V_{\text{soma}}}^{\Xi }=-1$$

## 3 Results

Details of the numerical solution techniques can be found in section S2 of the Supporting Information. In all figures, except for Fig 5, the following half‐lives are used for membrane fragments, free membrane aggregates, α‐syn monomers, and α‐syn aggregates: $T_{1/2,A,\mathrm{FR}}=2.70\times 10^{5}$ s, $T_{1/2,B,\mathrm{FR}}=1.35\times 10^{6}$ s, $T_{1/2,A,\mathrm{AS}}=5.76\times 10^{4}$ s, and $T_{1/2,B,\mathrm{AS}}=2.88\times 10^{5}$ s. The half‐lives of deposited membrane aggregates in the LB core and deposited α‐syn aggregates in the LB halo are assumed to be infinitely long. For numerical implementation, this is simulated by setting $T_{1/2,D,\mathrm{FR}}$ and $T_{1/2,D,\mathrm{FR}}$ to $10^{20}$ s. All other parameter values are as listed in Table S2 unless otherwise specified in the figure or its caption.

First, the numerical solutions for various values of $k_{1}$ were examined. The simulations assume that $k_{1,\mathrm{FR}}=k_{1,\mathrm{AS}}$. The molar concentrations of lipid membrane fragments and α‐syn monomers, $\left[A_{\mathrm{FR}}\right]$ and $\left[A_{\mathrm{AS}}\right]$, respectively, quickly reach equilibrium, remaining constant over time (Figure S1a). A similar pattern is observed for the molar concentrations of free lipid membrane aggregates in the LB core and free α‐syn aggregates, $\left[B_{\mathrm{FR}}\right]$ and $\left[B_{\mathrm{AS}}\right]$, respectively, which also reach their equilibrium values and become time‐independent (Figure S1b). The sudden changes in concentrations at $t=t_{\mathrm{LB},f}$ reflect the difference between the molar concentrations of membrane fragments and α‐syn.

The molar concentration of lipid membrane aggregates deposited in the LB core, $\left[D_{\mathrm{FR}}\right]$, remains low during the core's growth (Figure S2a), while the concentration of α‐syn aggregates deposited in the halo's fibrils, $\left[D_{\mathrm{AS}}\right]$, shows a linear increase over time (Figure S2a). The radii of both the growing LB core and halo follow an approximate cube root dependency on time (Figure S2b, the cube root dependency was first established in reference [16]). The noticeable increase in the LB core radius, $r_{\mathrm{LB}}$, over time, seen in Figure S2b, appears to contradict the nearly zero concentration of lipid membrane aggregates in the core, $\left[D_{\mathrm{FR}}\right]$, shown in Figure S2a. However, a detailed plot of the lipid membrane aggregates deposited into the LB core (Figure S3a) reveals that these concentrations increase with time, though their values are small, so their increase is not visible in Figure S2a. This is due to the large average molecular weight of membrane fragments (Table S2). The linear increase in $\left[D_{\mathrm{FR}}\right]$ over time (Figure S3a) leads to a corresponding linear increase in the LB core volume, resulting in the core radius growing proportionally to the cube root of time (Figure S3b). A smaller value of $k_{1}$ leads to a smaller LB radius (Figures S2b and S3b).

Next, the solutions to the problem for various values of $k_{2}$ are examined. The simulations assume that $k_{2,\mathrm{FR}}=k_{2,\mathrm{AS}}$. The molar concentrations of lipid membrane fragments, $\left[A_{\mathrm{FR}}\right]$, remain small (Figure S4a), while the molar concentrations of α‐syn monomers in the halo, $\left[A_{\mathrm{AS}}\right]$, reach different equilibrium values, depending on $k_{2,\mathrm{FR}}$ and $k_{2,\mathrm{AS}}$ (Figure S4a). Similarly, the molar concentrations of free lipid membrane aggregates and free α‐syn aggregates, $\left[B_{\mathrm{FR}}\right]$ and $\left[B_{\mathrm{AS}}\right]$, respectively, follow the same pattern (Figure S4b). Interestingly, as $k_{2,\mathrm{FR}}$ and $k_{2,\mathrm{AS}}$ increase, the concentration of α‐syn monomers in the halo decreases, whereas the concentration of free α‐syn aggregates in the halo increases.

The molar concentration of lipid membrane aggregates deposited into the LB core, $\left[D_{\mathrm{FR}}\right]$, remains small during the core's growth (Figure S5a), while the concentration of α‐syn aggregates deposited in the halo's fibrils, $\left[D_{\mathrm{AS}}\right]$, shows a linear increase over time (Figure S5a). The radii of the growing LB core and halo, $r_{\mathrm{LB}}$ and $r_{H}$, respectively, follow a cube root dependency with time (Figure S5b), with smaller values of $k_{2}$ corresponding to a smaller LB radius (Figures S5b).

The effects of the production rates of lipid membrane fragments and α‐syn monomers are similar to the effects of $k_{2}$. The concentrations of free α‐syn aggregates, $\left[B_{\mathrm{AS}}\right]$, reach different equilibrium levels depending on the value of $q_{\mathrm{AS}}$ (Figure S6b). Meanwhile, the concentration of α‐syn aggregates deposited in the halo's fibrils, $\left[D_{\mathrm{AS}}\right]$, increases linearly over time, with faster growth observed for larger $q_{\mathrm{AS}}$ values (Figure S7a). The radii of both the growing LB core and the halo, $r_{\mathrm{LB}}$ and $r_{H}$, expand approximately according to a cube root dependence with time, with faster growth for higher $q_{\mathrm{FR}}$ and $q_{\mathrm{AS}}$ values (Figure S7b).

**FIGURE 2:**
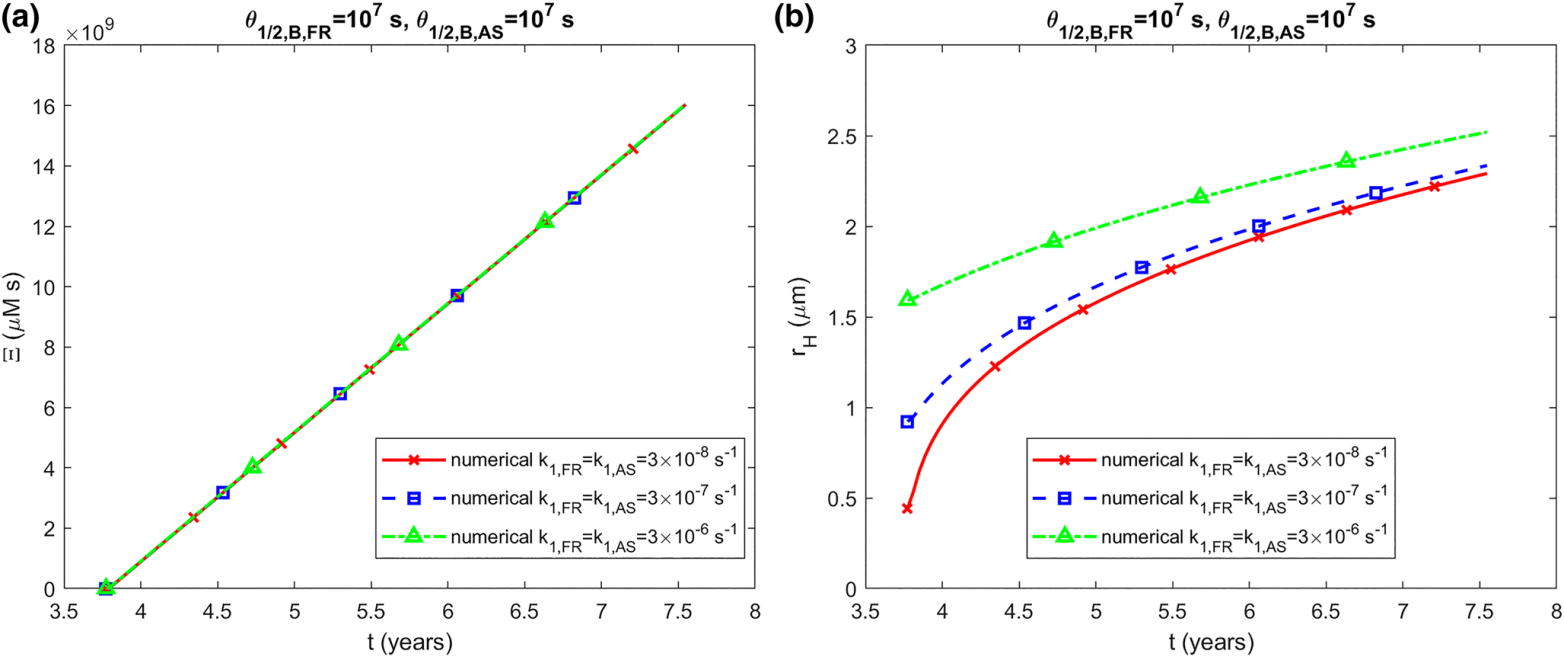
(a) Accumulated neurotoxicity of α‐syn oligomers, Ξ, versus time. (b) The radius of the growing LB halo, $r_{H}$, versus time. The results are presented for various values of $k_{1,\mathrm{FR}}$ and $k_{1,\mathrm{AS}}$. ($k_{2,\mathrm{FR}}=k_{2,\mathrm{AS}}=2\times 10^{-6}$ μM^−1^ s^−1^, $q_{\mathrm{FR}}=1.57\times 10^{-28}$ mol s^−1^, $q_{\mathrm{AS}}=1.47\times 10^{-21}$ mol s^−1^).

The accumulated neurotoxicity of α‐syn oligomers, Ξ, calculated using Equation (40), increases linearly over time and is independent of $k_{1}$ (Figure 2a). This linear increase occurs because the concentration of α‐syn oligomers, $\left[B_{\mathrm{AS}}\right]$, remains constant over time (Figure S1b) and $\int_{0}^{t}\mathrm{Cd}\hat{t}=\mathrm{Ct}$ if C is constant. While the radius of the LB halo, $r_{H}$, also increases over time, it does so nonlinearly (Figure 2b). The dependence of the halo radius on $k_{1}$ in Figure 2b may seem unexpected since $\left[D_{\mathrm{AS}}\right]$ in Figure S2 is independent of $k_{1}$, but the LB core radius is influenced by $k_{1}$ (Figure S2b), causing the halo to begin expanding from different initial radii that depend on the value of $k_{1}$.

**FIGURE 3:**
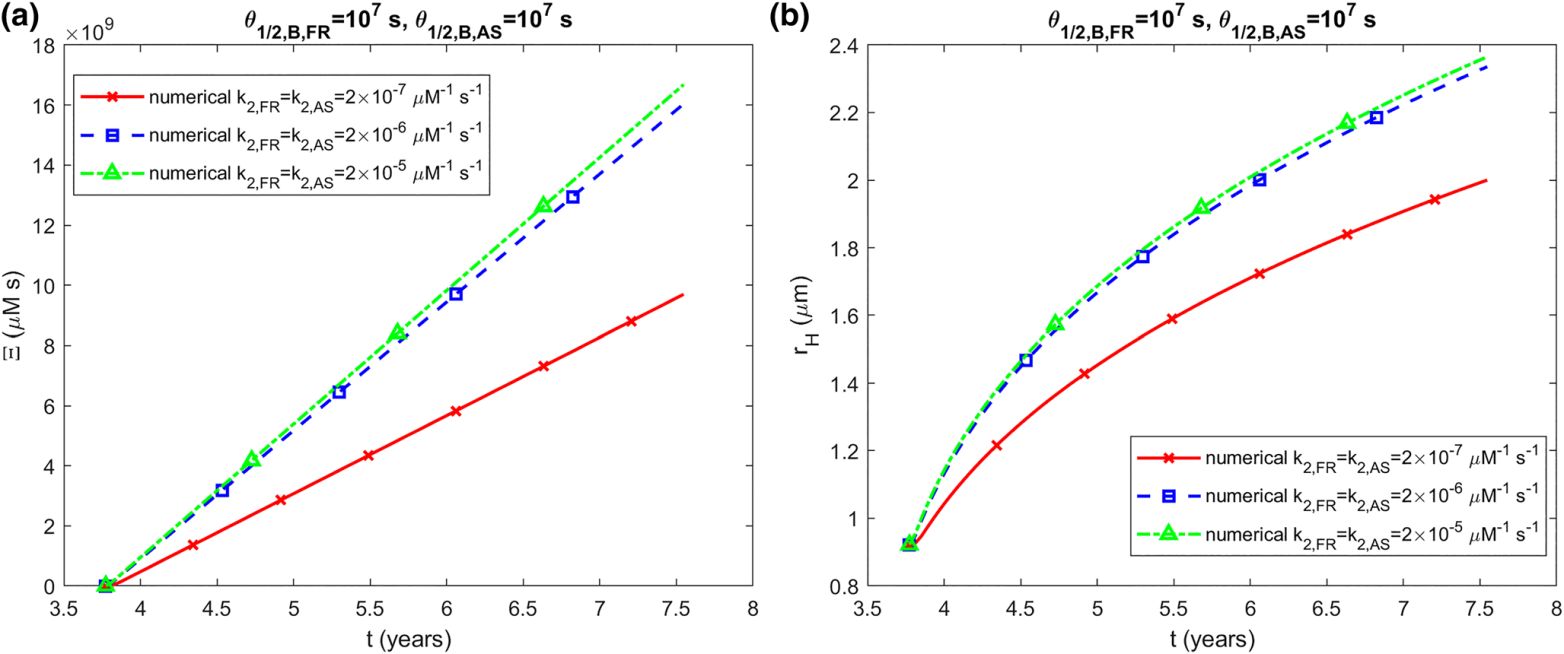
(a) Accumulated neurotoxicity of α‐syn oligomers, Ξ, versus time. (b) Radius of the growing LB halo, $r_{H}$, versus time. Results are displayed for different values of $k_{2,\mathrm{FR}}$ and $k_{2,\mathrm{AS}}$ ($k_{1,\mathrm{FR}}=k_{1,\mathrm{AS}}=3\times 10^{-7}$ s^−1^, $q_{\mathrm{FR}}=1.57\times 10^{-28}$ mol s^−1^, $q_{\mathrm{AS}}=1.47\times 10^{-21}$ mol s^−1^).

**FIGURE 4:**
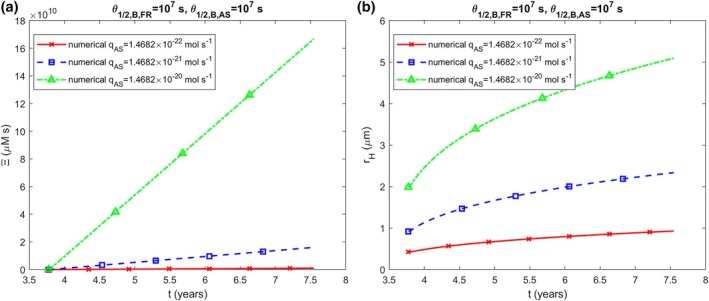
(a) Accumulated neurotoxicity of α‐syn oligomers, Ξ, versus time. (b) The radius of the expanding LB halo versus time. Results are presented for various values of $q_{\mathrm{FR}}$ and $q_{\mathrm{AS}}$. The following corresponding values were used for $q_{\mathrm{FR}}$: $q_{\mathrm{FR}}=1.57\times 10^{-29}$ mol s^−1^ was used for $q_{\mathrm{AS}}=1.47\times 10^{-22}$ mol s^−1^, $q_{\mathrm{FR}}=1.57\times 10^{-28}$ mol s^−1^ was used for $q_{\mathrm{AS}}=1.47\times 10^{-21}$ mol s^−1^, and $q_{\mathrm{FR}}=1.57\times 10^{-27}$ mol s^−1^ was used for $q_{\mathrm{AS}}=1.47\times 10^{-20}$ mol s^−1^. ($k_{1,\mathrm{FR}}=k_{1,\mathrm{AS}}=3\times 10^{-7}$ s^−1^, $k_{2,\mathrm{FR}}=k_{2,\mathrm{AS}}=2\times 10^{-6}$ μM^−1^ s^−1^).

**FIGURE 5:**
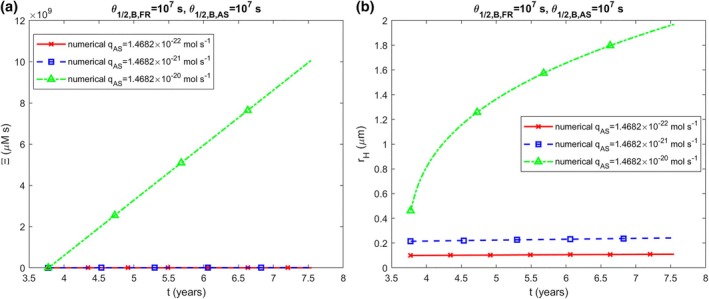
Similar to Figure 2, but with monomers and free aggregates having half‐lives reduced by an order of magnitude: $T_{1/2,A,\mathrm{FR}}=2.70\times 10^{4}$ s, $T_{1/2,B,\mathrm{FR}}=1.35\times 10^{5}$ s, $T_{1/2,A,\mathrm{AS}}=5.76\times 10^{3}$ s, $T_{1/2,B,\mathrm{AS}}=2.88\times 10^{4}$ s.

The accumulated neurotoxicity rises as $k_{2}$ increases (Figure 3a), with the halo radius showing a similar growth trend (Figure 3b). Likewise, the accumulated neurotoxicity also increases when the production rates of membrane fragments and α‐syn monomers, $q_{\mathrm{FR}}$ and $q_{\mathrm{AS}}$, are higher (Figure 4a). The halo radius also increases when $q_{\mathrm{FR}}$ and $q_{\mathrm{AS}}$ increase (Figure 4b). The scenario shown in Figure 4 was recalculated using half‐lives of monomers and free aggregates that are an order of magnitude shorter, corresponding to a tenfold increase in their degradation rates ($T_{1/2,A,\mathrm{FR}}=2.70\times 10^{4}$ s, $T_{1/2,B,\mathrm{FR}}=1.35\times 10^{5}$ s, $T_{1/2,A,\mathrm{AS}}=5.76\times 10^{3}$ s, $T_{1/2,B,\mathrm{AS}}=2.88\times 10^{4}$ s). The results indicate approximately 15 times lower neurotoxicity (Figure 5a) and a roughly 2.5 times smaller LB halo radius (Figure 5b).

The role of LBs in PD remains unclear, with researchers debating whether they merely sequester toxic α‐syn oligomers or are themselves harmful to neurons [12, 13, 14, 17, 48, 49]. If LBs simply act as storage for cytotoxic α‐syn species, one would expect their numbers to increase as the disease progresses. Surprisingly, this is not the case. As PD advances, the number of neurons containing LBs in the substantia nigra pars compacta does not rise; instead, neurons harboring LBs tend to die [15]. This observation points to a more active role for LBs in contributing to neurodegeneration. The increase in accumulated neurotoxicity of α‐syn aggregates may be the true cause of neuron death, with the size of LBs merely showing a correlation with this neurotoxicity.

**FIGURE 6:**
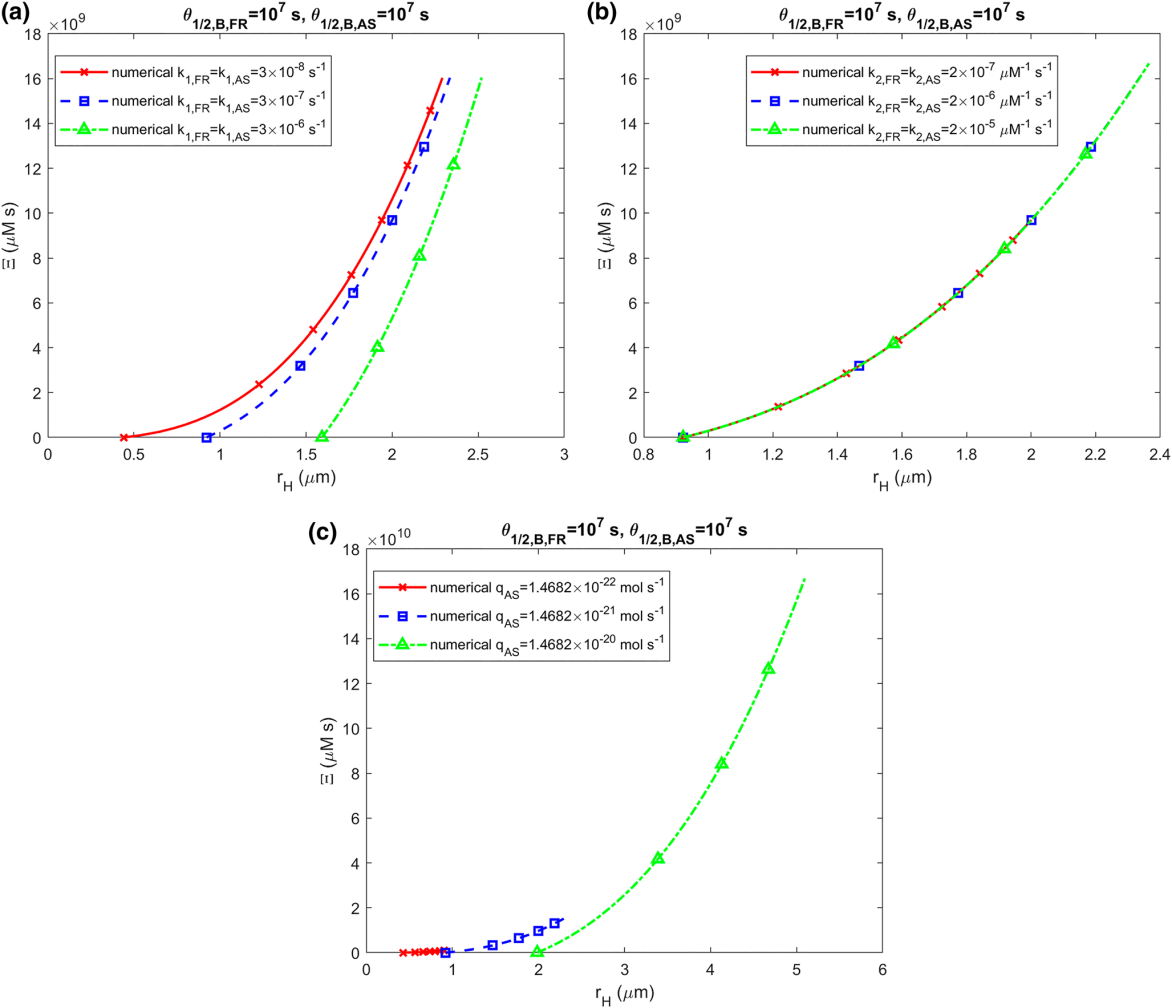
The accumulated neurotoxicity of α‐syn oligomers, Ξ, versus the radius of a growing LB halo, $r_{H}$, is shown for three different conditions: (a) varying nucleation rate constants, $k_{1,\mathrm{FR}}$ and $k_{1,\mathrm{AS}}$, (b) varying autocatalytic rate constants, $k_{2,\mathrm{FR}}$ and $k_{2,\mathrm{AS}}$, and (c) different production rates of membrane fragments and α‐syn monomers, $q_{\mathrm{FR}}$ and $q_{\mathrm{AS}}$. The following corresponding values were used for $q_{\mathrm{FR}}$: $q_{\mathrm{FR}}=1.57\times 10^{-29}$ mol s^−1^ was used for $q_{\mathrm{AS}}=1.47\times 10^{-22}$ mol s^−1^, $q_{\mathrm{FR}}=1.57\times 10^{-28}$ mol s^−1^ was used for $q_{\mathrm{AS}}=1.47\times 10^{-21}$ mol s^−1^, and $q_{\mathrm{FR}}=1.57\times 10^{-27}$ mol s^−1^ was used for $q_{\mathrm{AS}}=1.47\times 10^{-20}$ mol s^−1^. ($k_{1,\mathrm{FR}}=k_{1,\mathrm{AS}}=3\times 10^{-7}$ s^−1^, $k_{2,\mathrm{FR}}=k_{2,\mathrm{AS}}=2\times 10^{-6}$ μM^−1^ s^−1^).

Figure 6a–c suggests a correlation between accumulated neurotoxicity, Ξ, and the radius of the LB halo, $r_{H}$, such that as $r_{H}$ increases, Ξ also increases. Assuming that neurons die when the halo radius reaches its maximum value, the critical level of accumulated neurotoxicity can be roughly estimated from Figure 6a–c as $10^{10}$ μM·s. However, this is a preliminary estimate that requires validation through future experimental studies. It is important to note that the curves representing $\Xi \left(r_{H}\right)$ for small values of $q_{\mathrm{AS}}$ in Figure 6c terminate at lower $r_{H}$ values. This is because the halo radius does not grow significantly for small $q_{\mathrm{AS}}$ (Figures 4b and S7b).

**FIGURE 7:**
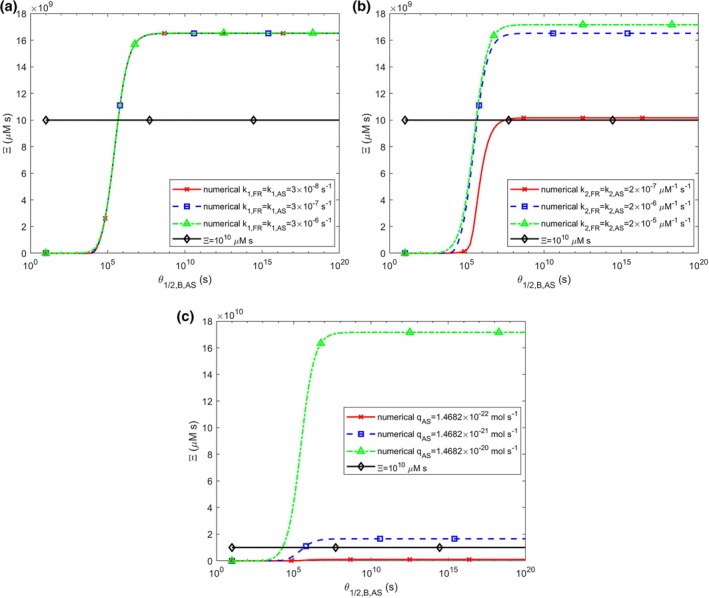
The accumulated neurotoxicity of α‐syn oligomers, Ξ, versus the half‐deposition time of free α‐syn aggregates into fibrils, $\theta_{1/2,B,\mathrm{AS}}$, for three different values of (a) nucleation rate constants, $k_{1,\mathrm{FR}}$ and $k_{1,\mathrm{AS}}$ ($k_{2,\mathrm{FR}}=k_{2,\mathrm{AS}}=2\times 10^{-6}$ μM^−1^ s^−1^, $q_{\mathrm{FR}}=1.57\times 10^{-28}$ mol s^−1^, $q_{\mathrm{AS}}=1.47\times 10^{-21}$ mol s^−1^), (b) autocatalytic rate constants, $k_{2,\mathrm{FR}}$ and $k_{2,\mathrm{AS}}$ ($k_{1,\mathrm{FR}}=k_{1,\mathrm{AS}}=3\times 10^{-7}$ s^−1^, $q_{\mathrm{FR}}=1.57\times 10^{-28}$ mol s^−1^, $q_{\mathrm{AS}}=1.47\times 10^{-21}$ mol s^−1^), (c) production rates of membrane fragments and α‐syn monomers, $q_{\mathrm{FR}}$ and $q_{\mathrm{AS}}$. The following corresponding values were used for $q_{\mathrm{FR}}$: $q_{\mathrm{FR}}=1.57\times 10^{-29}$ mol s^−1^ was used for $q_{\mathrm{AS}}=1.47\times 10^{-22}$ mol s^−1^, $q_{\mathrm{FR}}=1.57\times 10^{-28}$ mol s^−1^ was used for $q_{\mathrm{AS}}=1.47\times 10^{-21}$ mol s^−1^, and $q_{\mathrm{FR}}=1.57\times 10^{-27}$ mol s^−1^ was used for $q_{\mathrm{AS}}=1.47\times 10^{-20}$ mol s^−1^ ($k_{1,\mathrm{FR}}=k_{1,\mathrm{AS}}=3\times 10^{-7}$ s^−1^, $k_{2,\mathrm{FR}}=k_{2,\mathrm{AS}}=2\times 10^{-6}$ μM^−1^ s^−1^). An assumption that $\theta_{1/2,B,\mathrm{FR}}=\theta_{1/2,B,\mathrm{AS}}$ was used in the computer code.

When $\theta_{1/2,B,\mathrm{AS}}$ is small, α‐syn oligomers rapidly deposit into fibrils, resulting in zero accumulated neurotoxicity (Figure 7a–c). This supports the hypothesis that LBs may have a neuroprotective function [7]. The highest level of accumulated neurotoxicity occurs as $\theta_{1/2,B,\mathrm{AS}}\to \infty$ because, in this case, free α‐syn aggregates remain in the cytosol indefinitely, continuing to catalyze their own production from α‐syn monomers. The model suggests that deposition into LBs effectively removes α‐syn oligomers from the cytosol. Notably, some lines in Figure 7a–c cross the critical neurotoxicity threshold, $10^{10}$ μM s, when $\theta_{1/2,B,\mathrm{AS}}$ increases. This is an indication that these neurons will die.

The curves showing the dependence of Ξ on $\theta_{1/2,B,\mathrm{AS}}$ are unaffected by the kinetic constants related to nucleation processes, $k_{1,\mathrm{FR}}$ and $k_{1,\mathrm{AS}}$ (Figure 7a). However, Ξ increases as the kinetic constants of the autocatalytic process, $k_{2,\mathrm{FR}}$ and $k_{2,\mathrm{AS}}$, increase (Figure 7b), indicating that these autocatalytic constants have a greater impact on accumulated neurotoxicity than the nucleation constants, $k_{1,\mathrm{FR}}$ and $k_{1,\mathrm{AS}}$. Additionally, accumulated neurotoxicity increases rapidly with increased production rates of membrane fragments and α‐syn monomers, $q_{\mathrm{FR}}$ and $q_{\mathrm{AS}}$ (Figure 7c).

**FIGURE 8:**
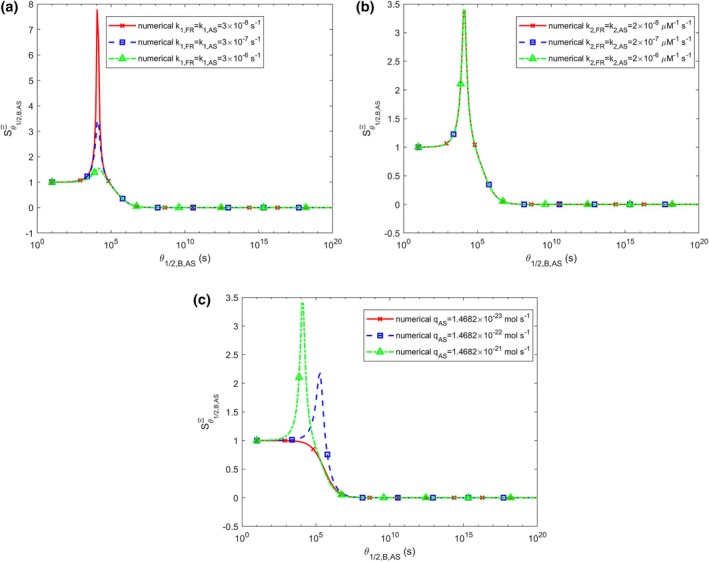
Sensitivity of accumulated neurotoxicity, Ξ, to the half‐deposition time of free α‐syn aggregates into fibrils, $\theta_{1/2,B,\mathrm{AS}}$, is examined for three different values of (a) the rate constants that describe the nucleation of membrane fragments and α‐syn aggregates, $k_{1,\mathrm{FR}}$ and $k_{1,\mathrm{AS}}$ ($k_{2,\mathrm{FR}}=k_{2,\mathrm{AS}}=2\times 10^{-6}$ μM^−1^ s^−1^, $q_{\mathrm{FR}}=1.57\times 10^{-28}$ mol s^−1^, $q_{\mathrm{AS}}=1.47\times 10^{-21}$ mol s^−1^), (b) the rate constants that describe the autocatalytic growth of membrane fragments and α‐syn aggregates, $k_{2,\mathrm{FR}}$ and $k_{2,\mathrm{AS}}$ ($k_{1,\mathrm{FR}}=k_{1,\mathrm{AS}}=3\times 10^{-7}$ s^−1^, $q_{\mathrm{FR}}=1.57\times 10^{-28}$ mol s^−1^, $q_{\mathrm{AS}}=1.47\times 10^{-21}$ mol s^−1^), (c) the production rate of α‐syn monomers, $q_{\mathrm{AS}}$ ($k_{1,\mathrm{FR}}=k_{1,\mathrm{AS}}=3\times 10^{-7}$ s^−1^, $k_{2,\mathrm{FR}}=k_{2,\mathrm{AS}}=2\times 10^{-6}$ μM^−1^ s^−1^). An assumption that $\theta_{1/2,B,\mathrm{FR}}=\theta_{1/2,B,\mathrm{AS}}$ was used in the computer code.

The sensitivity of accumulated neurotoxicity, Ξ, to the half‐deposition time of free α‐syn aggregates into fibrils, $\theta_{1/2,B,\mathrm{AS}}$, reveals sharp peaks in the transition region (Figure 8a–c). This transition occurs from a zero value of Ξ (when free α‐syn aggregates rapidly deposit into LBs) to the maximum value of Ξ at large $\theta_{1/2,B,\mathrm{AS}}$ (when free α‐syn aggregates remain in the cytosol and do not deposit into LBs) (Figure 7a–c). The location of the peak of $S_{\theta_{1/2,B,\mathrm{AS}}}^{\Xi }$ is unaffected by the values of $k_{1,\mathrm{FR}}$ and $k_{1,\mathrm{AS}}$, though the amplitude of the peak increases as $k_{1,\mathrm{AS}}$ decreases (Figure 8a). Both the amplitude and location of the peak of $S_{\theta_{1/2,B,\mathrm{AS}}}^{\Xi }$ remain independent of $k_{2,\mathrm{FR}}$ and $k_{2,\mathrm{AS}}$ (Figure 8b). The peak location of sensitivity shifts to smaller $\theta_{1/2,B,\mathrm{AS}}$ values as $q_{\mathrm{AS}}$ increases. Interestingly, for the smallest $q_{\mathrm{AS}}$ value, the peak of $S_{\theta_{1/2,B,\mathrm{AS}}}^{\Xi }$ disappears entirely (Figure 8c). Note that the numerically computed curves for $S_{\theta_{1/2,B,\mathrm{AS}}}^{\Xi }$ align with the analytical prediction, $S_{\theta_{1/2,B,\mathrm{AS}}}^{\Xi }=0$, given by Equation (41), as $\theta_{1/2,B,\mathrm{AS}}\to \infty$.

**FIGURE 9:**
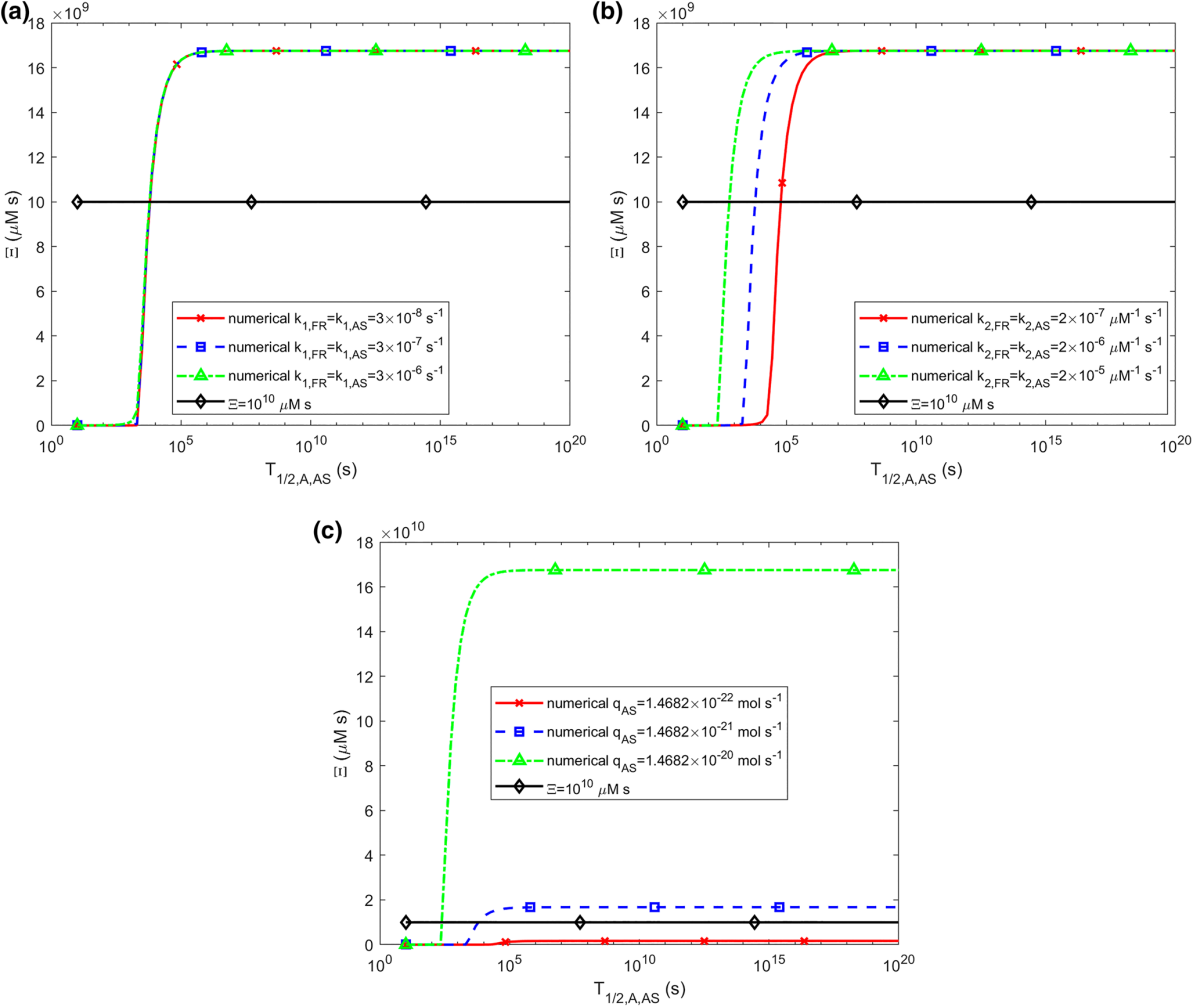
The accumulated neurotoxicity of α‐syn oligomers, Ξ, versus the half‐life of α‐syn monomers, $T_{1/2,A,\mathrm{AS}}$, for three different values of (a) nucleation rate constants, $k_{1,\mathrm{FR}}$ and $k_{1,\mathrm{AS}}$ ($k_{2,\mathrm{FR}}=k_{2,\mathrm{AS}}=2\times 10^{-6}$ μM^−1^ s^−1^, $q_{\mathrm{FR}}=1.57\times 10^{-28}$ mol s^−1^, $q_{\mathrm{AS}}=1.47\times 10^{-21}$ mol s^−1^), (b) autocatalytic rate constants, $k_{2,\mathrm{FR}}$ and $k_{2,\mathrm{AS}}$ ($k_{1,\mathrm{FR}}=k_{1,\mathrm{AS}}=3\times 10^{-7}$ s^−1^, $q_{\mathrm{FR}}=1.57\times 10^{-28}$ mol s^−1^, $q_{\mathrm{AS}}=1.47\times 10^{-21}$ mol s^−1^), (c) production rates of membrane fragments and α‐syn monomers, $q_{\mathrm{FR}}$ and $q_{\mathrm{AS}}$. The following corresponding values were used for $q_{\mathrm{FR}}$: $q_{\mathrm{FR}}=1.57\times 10^{-29}$ mol s^−1^ was used for $q_{\mathrm{AS}}=1.47\times 10^{-22}$ mol s^−1^, $q_{\mathrm{FR}}=1.57\times 10^{-28}$ mol s^−1^ was used for $q_{\mathrm{AS}}=1.47\times 10^{-21}$ mol s^−1^, and $q_{\mathrm{FR}}=1.57\times 10^{-27}$ mol s^−1^ was used for $q_{\mathrm{AS}}=1.47\times 10^{-20}$ mol s^−1^ ($k_{1,\mathrm{FR}}=k_{1,\mathrm{AS}}=3\times 10^{-7}$ s^−1^, $k_{2,\mathrm{FR}}=k_{2,\mathrm{AS}}=2\times 10^{-6}$ μM^−1^ s^−1^). $T_{1/2,B,\mathrm{AS}}$, $T_{1/2,A,\mathrm{FR}}$, and $T_{1/2,B,\mathrm{FR}}$ were kept at their values given in Table S2.

Age‐related declines in α‐syn degradation mechanisms have been proposed as a contributing factor to α‐syn aggregation [4]. Malfunctions in these degradation pathways would result in longer half‐lives for α‐syn monomers and free aggregates, $T_{1/2,A,\mathrm{AS}}$ and $T_{1/2,B,\mathrm{AS}}$, respectively. An increase in $T_{1/2,A,\mathrm{AS}}$ leads to an S‐shaped rise in accumulated neurotoxicity, Ξ (Figure 9). Importantly, an increase in $T_{1/2,A,\mathrm{AS}}$ causes the accumulated neurotoxicity to exceed the critical threshold of $10^{10}$ μM·s, indicating that the affected neurons will undergo cell death. This increase in Ξ is independent of the nucleation rate constants, $k_{1,\mathrm{FR}}$ and $k_{1,\mathrm{AS}}$ (Figure 9a). Higher values of the rate constants associated with autocatalytic growth, $k_{2,\mathrm{FR}}$ and $k_{2,\mathrm{AS}}$, cause the rise in Ξ to begin at smaller values of $T_{1/2,A,\mathrm{AS}}$. However, the asymptotic value of Ξ for large $T_{1/2,A,\mathrm{AS}}$ remains unaffected by $k_{2,\mathrm{FR}}$ and $k_{2,\mathrm{AS}}$ (Figure 9b). Additionally, the asymptotic values of Ξ for large $T_{1/2,A,\mathrm{AS}}$ increase with an increase in the production rate of α‐syn monomers, $q_{\mathrm{AS}}$ (Figure 9c).

**FIGURE 10:**
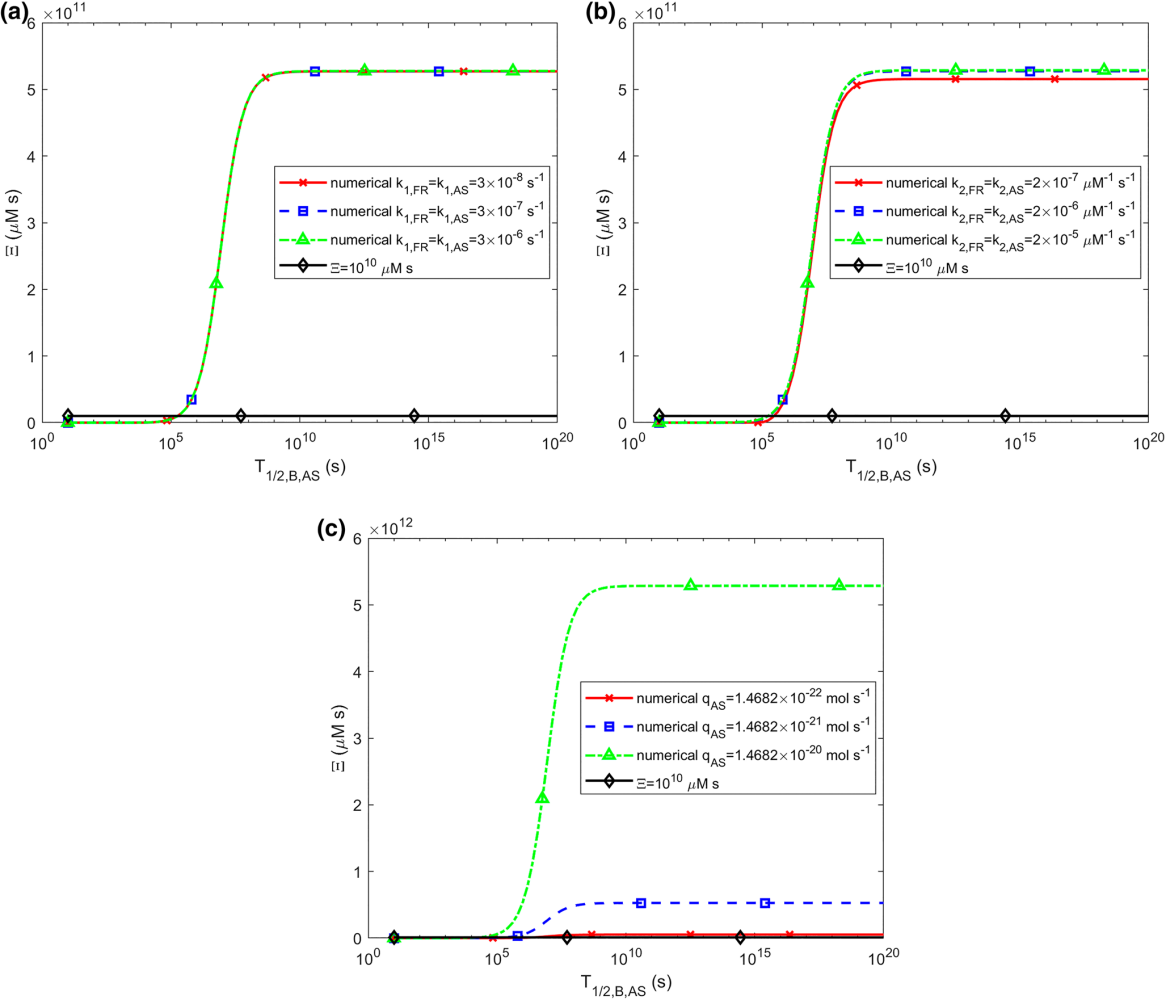
The accumulated neurotoxicity of α‐syn oligomers, Ξ, versus the half‐life of free α‐syn aggregates, $T_{1/2,B,\mathrm{AS}}$, for three different values of (a) nucleation rate constants, $k_{1,\mathrm{FR}}$ and $k_{1,\mathrm{AS}}$ ($k_{2,\mathrm{FR}}=k_{2,\mathrm{AS}}=2\times 10^{-6}$ μM^−1^ s^−1^, $q_{\mathrm{FR}}=1.57\times 10^{-28}$ mol s^−1^, $q_{\mathrm{AS}}=1.47\times 10^{-21}$ mol s^−1^), (b) autocatalytic rate constants, $k_{2,\mathrm{FR}}$ and $k_{2,\mathrm{AS}}$ ($k_{1,\mathrm{FR}}=k_{1,\mathrm{AS}}=3\times 10^{-7}$ s^−1^, $q_{\mathrm{FR}}=1.57\times 10^{-28}$ mol s^−1^, $q_{\mathrm{AS}}=1.47\times 10^{-21}$ mol s^−1^), (c) production rates of membrane fragments and α‐syn monomers, $q_{\mathrm{FR}}$ and $q_{\mathrm{AS}}$. The following corresponding values were used for $q_{\mathrm{FR}}$: $q_{\mathrm{FR}}=1.57\times 10^{-29}$ mol s^−1^ was used for $q_{\mathrm{AS}}=1.47\times 10^{-22}$ mol s^−1^, $q_{\mathrm{FR}}=1.57\times 10^{-28}$ mol s^−1^ was used for $q_{\mathrm{AS}}=1.47\times 10^{-21}$ mol s^−1^, and $q_{\mathrm{FR}}=1.57\times 10^{-27}$ mol s^−1^ was used for $q_{\mathrm{AS}}=1.47\times 10^{-20}$ mol s^−1^ ($k_{1,\mathrm{FR}}=k_{1,\mathrm{AS}}=3\times 10^{-7}$ s^−1^, $k_{2,\mathrm{FR}}=k_{2,\mathrm{AS}}=2\times 10^{-6}$ μM^−1^ s^−1^). $T_{1/2,A,\mathrm{AS}}$, $T_{1/2,A,\mathrm{FR}}$, and $T_{1/2,B,\mathrm{FR}}$ were kept at their values given in Table S2.

The accumulated neurotoxicity, Ξ, also shows an S‐shaped increase with the increase in $T_{1/2,B,\mathrm{AS}}$ (Figure 10). The increase in $T_{1/2,B,\mathrm{AS}}$ results in the accumulated neurotoxicity exceeding the critical threshold of $10^{10}$ μM·s. This increase is independent of the values of $k_{1,\mathrm{FR}}$ and $k_{1,\mathrm{AS}}$ (Figure 10a). There is a minimal dependence of Ξ on the growth of $k_{2,\mathrm{FR}}$ and $k_{2,\mathrm{AS}}$ (Figure 10b), with smaller values of $k_{2,\mathrm{FR}}$ and $k_{2,\mathrm{AS}}$ resulting in a slightly lower asymptotic value of Ξ as $T_{1/2,B,\mathrm{AS}}\to \infty$. A larger value of $q_{\mathrm{AS}}$ leads to a higher asymptotic value as $T_{1/2,B,\mathrm{AS}}\to \infty$ (Figure 10c).

The sensitivity of accumulated neurotoxicity, Ξ, to the half‐life of α‐syn monomers, $T_{1/2,A,\mathrm{AS}}$, displays a peak at the point where $\Xi \left(T_{1/2,A,\mathrm{AS}}\right)$ in Figure 9 transitions from zero to a high value. The location of these peaks is independent of $k_{1,\mathrm{FR}}$ and $k_{1,\mathrm{AS}}$, although their amplitudes are influenced by $k_{1,\mathrm{FR}}$ and $k_{1,\mathrm{AS}}$ (Figure S8). The sensitivity peak of Ξ shifts to smaller values of $T_{1/2,A,\mathrm{AS}}$ when $k_{2,\mathrm{FR}}$ and $k_{2,\mathrm{AS}}$ increase (Figure S8b), which aligns with the results shown in Figure 9b. Additionally, increasing $q_{\mathrm{AS}}$ also shifts the sensitivity peak of $S_{T_{1/2,A,\mathrm{AS}}}^{\Xi }$ toward smaller values of $T_{1/2,A,\mathrm{AS}}$ (Figure S8c), suggesting that for higher production rates of α‐syn monomers, the transition from zero to large values of Ξ occurs at smaller $T_{1/2,A,\mathrm{AS}}$, consistent with the results in Figure 9c. Intriguingly, the curves representing $S_{T_{1/2,A,\mathrm{AS}}}^{\Xi }$ in Figure S8b for three different values of $k_{2,\mathrm{AS}}$ (differing by an order of magnitude) coincide with the curves in Figure S8c for three different values of $q_{\mathrm{AS}}$ (also differing by an order of magnitude). This indicates that $S_{T_{1/2,A,\mathrm{AS}}}^{\Xi }$ responds in a similar manner to changes in both $k_{2,\mathrm{AS}}$ and $q_{\mathrm{AS}}$.

The position of the peak sensitivity of accumulated neurotoxicity, Ξ, to the half‐life of free α‐syn aggregates, $T_{1/2,B,\mathrm{AS}}$, remains unaffected by changes in $k_{1,\mathrm{FR}}$ and $k_{1,\mathrm{AS}}$. However, the peak amplitude increases as $k_{1,\mathrm{FR}}$ and $k_{1,\mathrm{AS}}$ decrease (Figure S9a). The position of the peak of $S_{T_{1/2,B,\mathrm{AS}}}^{\Xi }$ shifts to lower values of $T_{1/2,B,\mathrm{AS}}$ when $k_{2,\mathrm{FR}}$ and $k_{2,\mathrm{AS}}$ increase (Figure S9b). Similarly, an increase in the production rate of α‐syn monomers, $q_{\mathrm{AS}}$, also causes the position of the peak of $S_{T_{1/2,B,\mathrm{AS}}}^{\Xi }$ to shift to smaller values of $T_{1/2,B,\mathrm{AS}}$ (Figure S9c). The curves illustrating $S_{T_{1/2,B,\mathrm{AS}}}^{\Xi }$ in Figure S9b for three different values of $k_{2,\mathrm{AS}}$ (differing by an order of magnitude) are identical to those in Figure 9c for three different values of $q_{\mathrm{AS}}$ (also differing by an order of magnitude). This suggests that $S_{T_{1/2,B,\mathrm{AS}}}^{\Xi }$ exhibits an identical response to variations in both $k_{2,\mathrm{AS}}$ and $q_{\mathrm{AS}}$.

## 4 Discussion, Limitations of the Model, and Future Directions

The numerical investigation indicates that, for physiologically relevant parameter values, the concentration of free α‐syn oligomers remains constant over time (Figures S1b, S4b, and S6b). Since the accumulated neurotoxicity of α‐syn oligomers is defined as the integral of their concentration over time, the accumulated neurotoxicity increases linearly with time (Figures 2a, 3a and 4a). Assuming that neuron death occurs when the radius of the LB halo becomes sufficiently large, the critical value of accumulated neurotoxicity is estimated to be around $10^{10}$ μM·s.

The concentration of α‐syn aggregates deposited in the LB halo also increases linearly over time (Figures S2a, S5a, and S7a), leading to a linear increase in the volume of the LB halo. Consequently, the halo radius increases in proportion to the cube root of time (Figures S2b, S5b, and S7b).

Accumulated neurotoxicity increases with an increase in the α‐syn monomer production rate in the soma (Figure 4a). Shortening the half‐lives of monomers and free aggregates leads to a reduction in accumulated neurotoxicity (Figure 5a). Accordingly, reducing the production rate of α‐syn monomers or accelerating their degradation could help slow the accumulation of neurotoxicity.

The results shown in Figure 6 suggest a strong correlation between accumulated neurotoxicity and the radius of the LB halo. This could explain why neurons die when the LBs they contain grow large [15]. The underlying cause may not be the size of the LB itself but rather the high level of accumulated neurotoxicity, which happens to be correlated with the LB size.

Rapid deposition of α‐syn oligomers into fibrils results in zero accumulated neurotoxicity (Figure 7), so LBs may have a neuroprotective role [7]. Conversely, the highest neurotoxicity occurs when oligomers deposit slowly, remaining in the cytosol and catalyzing their own production from monomers. The model suggests that LB formation helps clear α‐syn oligomers from the cytosol, reducing their toxic effects.

It is important to note that although Equation (40) represents a simple time integration, accumulated neurotoxicity is significantly more complex. This complexity arises because the integrated quantity, the concentration of α‐syn oligomers, is governed by a nonlinear equation, as α‐syn oligomers can catalyze their own production. For example, accumulated neurotoxicity is highly sensitive to the half‐deposition time of free α‐syn aggregates into fibrils. A pronounced peak in sensitivity appears in the range where accumulated neurotoxicity shifts from negligible (at short half‐deposition times) to high levels (at longer times); compare Figures 7 and 8. The exact position of this peak varies depending on other parameter values.

The findings demonstrate that increased half‐lives of α‐syn monomers and aggregates cause an S‐shaped increase in accumulated neurotoxicity, which eventually surpasses the critical threshold and leads to neuron death. This aligns with the widely accepted hypothesis in biomedical literature that disruptions in protein degradation pathways may contribute to the progression of PD [22].

α‐syn oligomers can form in two ways: either during the self‐assembly of α‐syn monomers or through the release from already formed fibrillar species [23]. Future research should expand the model to include the potential release of α‐syn oligomers from fibrillar species.

Since misfolded, aggregated species of α‐syn are known for their ability to catalyze their own production and propagate to connected neurons (acting as propagons, as noted in reference [7]), it would be important to develop a model that combines the current framework for α‐syn aggregation with one that describes the transport of α‐syn aggregates. These aggregates could act as seeds for the formation of new α‐syn aggregates in neighboring neurons. The accumulated toxicity parameter, which accounts for cumulative time‐dependent damage, can also be applied beyond the neural domain.

## Conflicts of Interest

The author declares no conflicts of interest.

## Supporting information


Data S1.

## Data Availability

Data sharing not applicable to this article as no datasets were generated or analysed during the current study.
