# Supplementary material for: Criterion for Assessing Accumulated Neurotoxicity of Alpha‐Synuclein Oligomers in Parkinson's Disease
Source: Int J Numer Method Biomed Eng. 2025 Apr 28;41(4):e70027. doi: 10.1002/cnm.70027 (PMC12036748; doi:10.1002/cnm.70027)
Supplement: Supplementary file 1 — Data S1. [file CNM-41-e70027-s001.pdf]

Criterion for Assessing Accumulated Neurotoxicity of Alpha-Synuclein Oligomers in Parkinson's Disease

Andrey V. Kuznetsov

Department of Mechanical and Aerospace Engineering, North Carolina State University,

Raleigh, NC 27695-7910, USA; e-mail: avkuznet@ncsu.edu

Supplemental Materials

S1. Dimensionless parameter for accumulated neurotoxicity of α-syn oligomers

The accumulated neurotoxicity of α-syn oligomers, expressed in Eq. (40), can be reformulated into a dimensionless quantity as follows:

$$\Xi^*\left(t^*\right)=\int_{t^*_{LB,f}}^{t^*}\left[B^*_{AS}\right]\left(\hat{t}^*\right)d\hat{t}^* \tag{S1}$$

where

$$\Xi^*=\Xi k_{2,AS} \tag{S2}$$

$$t^*=t k_{1,AS} \tag{S3}$$

and

$$t^*_{LB,f}=t_{LB,f}\, k_{1,AS} \tag{S4}$$

S2. Numerical Solution

The system of equations, Eqs. (3)-(6) and (22)-(25), was solved using MATLAB's ODE45 solver (MATLAB R2020b, MathWorks, Natick, MA, USA). To ensure high precision, the relative and absolute tolerance parameters, RelTol and AbsTol, were set to 1e-10.

S3. Supplementary tables

Table S1. Dependent variables in the model.

Symbol	Definition	Units
$\left[A_{FR}\right]$	Molar concentration of all fragments within the soma that can contribute to the formation of the LB core—specifically, lipid membrane fragments, damaged organelles, lysosomes, and dysfunctional mitochondria	μM
$\left[B_{FR}\right]$	Molar concentration of free lipid membrane aggregates present in the soma	μM
$\left[D_{FR}\right]$	Molar concentration of lipid membrane aggregates that have been deposited into the LB core	μM
$\left[A_{AS}\right]$	Molar concentration of α-syn monomers present in the soma	μM
$\left[B_{AS}\right]$	Molar concentration of unbound α-syn aggregates in the soma	μM
$\left[D_{AS}\right]$	Molar concentration of α-syn aggregates that have been deposited into fibrils within the LB halo	μM
r_{LB}	Radius of the growing LB core	μm
r_H	Radius of the growing LB halo	μm

Table S2. Model parameters and their estimated values.

Symbol	Definition	Units	Value or range	Reference or estimation method	Value(s) used in computations
a_{21}	Conversion factor from $\frac{\text{mol}}{\mu\text{m}^3}$ to μM	$\frac{\mu\text{M}\,\mu\text{m}^3}{\text{mol}}$	10^{21}		10^{21}

$[A_{FR}]_0$	Initial concentration of lipid membrane fragments in the soma	μM			0 [a]
$[A_{AS}]_0$	Initial concentration of α-syn monomers in the soma	μM			0 [a]
D_{soma}	Diameter of the soma	μm	20 [b]		20
D_{LB}	Diameter of the LB, including the halo	μm	8-30 [c]	[17]	
$k_{1,FR}$	Rate constant for the first pseudoelementary step in the F-W model, which represents the nucleation of aggregates formed from lipid membrane fragments in the soma	s^{-1}			3×10^{-7} [d]
$k_{2,FR}$	Rate constant for the second pseudoelementary step in the F-W model, which characterizes the autocatalytic growth of lipid membrane	$\mu M^{-1}\ s^{-1}$			2×10^{-6} [e]

	fragment aggregates in the soma				
$k_{1,AS}$	Rate constant for the first pseudoelementary step in the F-W model, which describes the nucleation of free α-syn aggregates	s^{-1}	2.78×10^{-7} - 9.44×10^{-5} [f]	[31]	3×10^{-7}
$k_{2,AS}$	Rate constant for the second pseudoelementary step in the F-W model, which describes the autocatalytic growth of free α-syn aggregates through the addition of α-syn monomers in the soma	$\mu M^{-1}\ s^{-1}$	1.19×10^{-6} - 5.00×10^{-6} [g]	[31]	2×10^{-6}
MW_{FR}	Average molecular weight of a membrane fragment, defined as the total weight of all atoms within the fragment	$g\ mol^{-1}$	1.94×10^{10} [h]	[32]	1.94×10^{10}
MW_{AS}	Molecular weight of a single α-syn monomer	$g\ mol^{-1}$	1.45×10^{4} [i]	[33]	1.45×10^{4}

N_A	Avogadro's number	mol^{-1}	6.022×10^{23}		6.022×10^{23}
q_{FR}	Rate of production of lipid membrane fragments in the soma	mol s^{-1}	1.57×10^{-28} [j]	Estimated using Eq. (21)	1.57×10^{-28}
q_{AS}	Rate of production of α-syn monomers in the soma	mol s^{-1}	1.47×10^{-21} [k]	Estimated using Eq. (39)	1.47×10^{-21}
$t_{LB,f}$	Duration of LB core growth	s	1.19×10^{8} [l]	[34,48]	1.19×10^{8}
$t_{H,f}$	Duration of the growth period for the entire LB, including both the core and halo	s	2.38×10^{8} [m]	[34,48]	2.38×10^{8}
$T_{1/2,A,FR}$	Half-life of lipid membrane fragments, including damaged organelles, lysosomes, and damaged mitochondria, generally any fragments that could constitute the LB core	s	2.70×10^{5} [n]	[35]	2.70×10^{5}
$T_{1/2,B,FR}$	Half-life of free aggregated lipid membrane fragments,	s	1.35×10^{6} [o]		1.35×10^{6}

	not yet deposited into LBs				
$T_{1/2,D,FR}$	Half-life of lipid membrane fragments deposited into the LB core	s			10^{20}
$T_{1/2,A,AS}$	Half-life of α-syn monomers in the soma	s	5.76×10^{4} [p]	[36]	5.76×10^{4}
$T_{1/2,B,AS}$	Half-life of free α-syn aggregates in the soma	s	2.88×10^{5} [q]		2.88×10^{5}
$T_{1/2,D,AS}$	Half-life of α-syn aggregates embedded in the halo of an LB	s			10^{20}
V_{soma}	Volume of the soma	μm^3	4.19×10^{3} [r]		4.19×10^{3}
ρ_{LB}	Density of the LB, the same value is used for the core and halo	$g/\mu m^3$	1.35×10^{-12} [s]	[37]	1.35×10^{-12}
$\theta_{1/2,B,FR}$	Half-deposition time for free lipid membrane aggregates into the core of an LB, which is the duration needed for 50% of the aggregates in the cytosol to become	s			10^{7} [t]

	incorporated into the LB core				
$\theta_{1/2,B,AS}$	Half-deposition time of free α-syn aggregates into fibrils, which is the duration required for 50% of the aggregates in the cytosol to be incorporated into the α-syn fibrils	s			10^{7} [t]
Ξ_{crit}	Critical threshold of accumulated neurotoxicity caused by α-syn oligomers, which is the level at which the toxic effects of α-syn oligomers become severe enough to cause neuron death	μM·s	10^{10}	See the discussion of Fig. 5	10^{10}

[a] Since the formation of LBs is predominantly regulated by the rates at which lipid membrane fragments and α-syn monomers are produced and degraded, the effects of initial concentrations of these substances become negligible over extended periods. Therefore, both the initial concentrations of lipid membrane fragments and α-syn monomers are set to zero.

[b] A typical neuron with a soma diameter of 20 μm was used for the analysis.

[c] LBs are spherical structures with diameters ranging from 8 to 30 μm [17]. While this parameter is not directly utilized in the model, it is included in the table to estimate the radii of the LB core and halo, $r_{LB,f}$ and $r_{H,f}$, respectively. These radii are necessary for calculating q_{FR} and q_{AS} using Eqs. (21) and (39).

[d] Due to the lack of published data on the kinetic rates for the aggregation of lipid membrane fragments in the F-W model, the value of $k_{1,FR}$ was assumed to be equal to $k_{1,AS}$.

[e] In the absence of published data on the kinetic rates for lipid membrane fragment aggregation in the F-W model, $k_{2,FR}$ was assumed to be equal to $k_{2,AS}$.

[f] 0.001-0.34 h^{-1} [31].

[g] 0.0043-0.018 μM^{-1} h^{-1} [31].

[h] 14,460.16 dalton [33].

[i] Ref. [13] described LBs as consisting of a crowded environment composed of membrane fragments, mitochondria, and vesicular structures. According to ref. [32], a single mitochondrion has an estimated mass of 3.22×10^{-13} g, equivalent to 1.94×10^{11} dalton. The images presented in ref. [13] indicate that mitochondria are among the larger components within LBs. A representative mass of membranous inclusions within an LB was approximated to be one-tenth of that of a mitochondrion, or 1.94×10^{10} dalton.

[j] Eq. (21) was employed to estimate q_{FR}. The estimation was based on the following parameter values: $r_{LB,f} = 4$ μm, $\rho_{LB} = 1.35 \times 10^{-12}$ g/μm^3, $MW_{FR} = 1.94 \times 10^{10}$ g mol^{-1}, $t_{LB,f} = 1.19 \times 10^{8}$ s. This led to 1.57×10^{-28} mol s^{-1}.

[k] Eq. (39) was utilized to estimate q_{AS}, using the following parameter values: $r_{LB,f} = 4$ μm, $r_{H,f} = 8$ μm, $MW_{AS} = 1.45 \times 10^{4}$ g mol^{-1}, $\rho_{LB} = 1.35 \times 10^{-12}$ g/μm^3, $t_{LB,f} = 1.19 \times 10^{8}$ s, $t_{H,f} = 2.38 \times 10^{8}$ s. This led to 1.47×10^{-21} mol s^{-1}.

[l] The assumption is that the core of an LB grows for half of its total lifespan ($t_{LB,f} = t_{H,f}/2$), while the halo undergoes growth during the remaining half. According to footnote "m" in Table S2, $t_{LB,f} = 7.5$ years/2=3.75 years.

[m] Ref. [34] estimated the lifespan of an LB to be around 6 months, while ref. [48] proposed a lifespan of 7.5 years. For the purposes of this model, a lifespan of $t_{H,f} = 7.5$ years was assumed. It was further assumed that the growth of the LB halo begins at $t = t_{LB,f}$ and continues until $t = t_{H,f} = 2t_{LB,f}$, the point at which neuronal death occurs.

[n] According to ref. [35], membrane proteins have half-lives between 75 and 113 hours, while membrane lipids exhibit shorter half-lives, approximately 10% to 30% less than proteins. Based on this data, the half-life of membrane fragments was estimated at 75 hours.

[o] It was assumed that the half-life of aggregated lipid membrane fragments is five times greater than that of their unaggregated forms.

[p] Ref. [36] estimated the half-life of α-syn monomers to be 16 hours. This degradation occurs via both proteasomal and autophagic pathways [38].

[q] Ref. [39] reported that α-syn aggregates have a much longer half-life compared to their monomeric counterparts. In this study, it was assumed that the half-life of α-syn aggregates is five times greater than that of monomers.

[r] The soma is assumed to have a diameter of 20 μm.

[s] A widely accepted value for protein density, regardless of protein type, is 1.35 g/cm^3 [37].

[t] Since the deposition rates of free lipid membrane aggregates into the LB core and free α-syn aggregates into fibrils are currently unknown, a broad range of half-deposition times for both types of aggregates was explored in Figs. 7 and 8.

S4. Supplemental figures

(a)

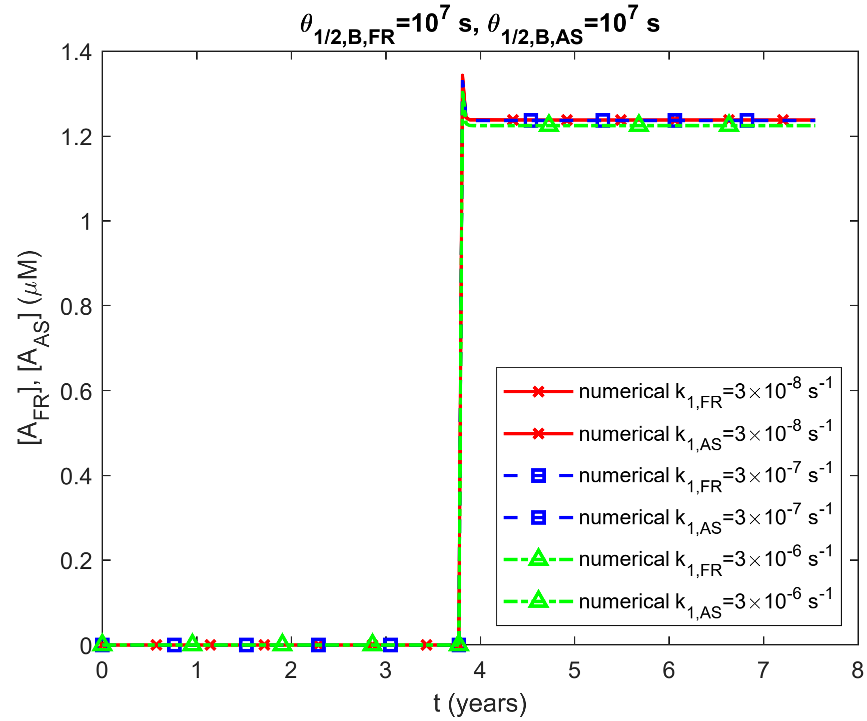

$\theta_{1/2,B,FR}$=10^7 s, $\theta_{1/2,B,AS}$=10^7 s
[A_FR], [A_AS] (μM)
t (years)
numerical k_1,FR=3×10^-8 s^-1
numerical k_1,AS=3×10^-8 s^-1
numerical k_1,FR=3×10^-7 s^-1
numerical k_1,AS=3×10^-7 s^-1
numerical k_1,FR=3×10^-6 s^-1
numerical k_1,AS=3×10^-6 s^-1

(b)

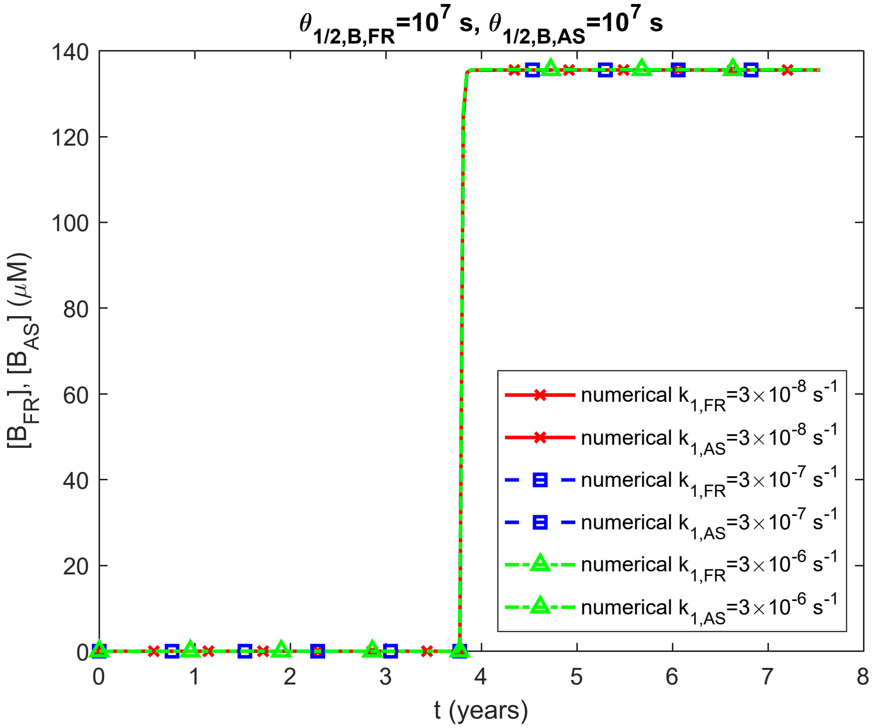

$\theta_{1/2,B,FR}$=10^7 s, $\theta_{1/2,B,AS}$=10^7 s
[B_FR], [B_AS] (μM)
t (years)
numerical k_1,FR=3×10^-8 s^-1
numerical k_1,AS=3×10^-8 s^-1
numerical k_1,FR=3×10^-7 s^-1
numerical k_1,AS=3×10^-7 s^-1
numerical k_1,FR=3×10^-6 s^-1
numerical k_1,AS=3×10^-6 s^-1

Fig. S1. (a) Molar concentrations of lipid membrane fragments and α-syn monomers, $\left[A_{FR}\right]$ and $\left[A_{AS}\right]$, respectively, for different values of $k_{1,FR}$ and $k_{1,AS}$ vs time. (b) Molar concentrations of free lipid membrane aggregates and free α-syn aggregates, $\left[B_{FR}\right]$ and $\left[B_{AS}\right]$, respectively, for different values of $k_{1,FR}$ and $k_{1,AS}$ vs time. ($k_{2,FR}=k_{2,AS}=2\times10^{-6}$ μM^{-1} s^{-1}, $q_{FR}=1.57\times10^{-28}$ mol s^{-1}, $q_{AS}=1.47\times10^{-21}$ mol s^{-1}.)

(a)

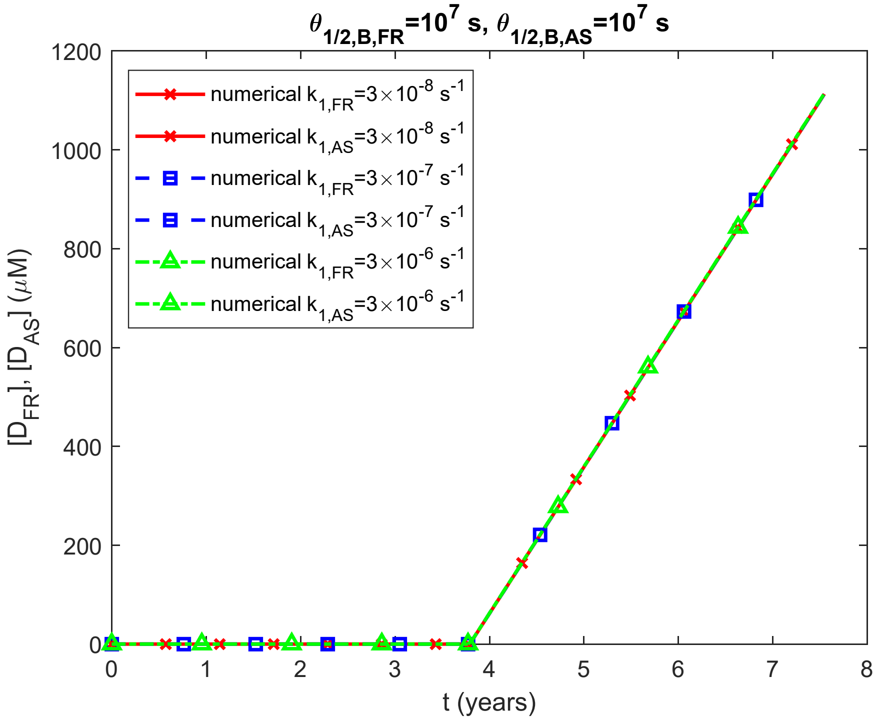

(b)

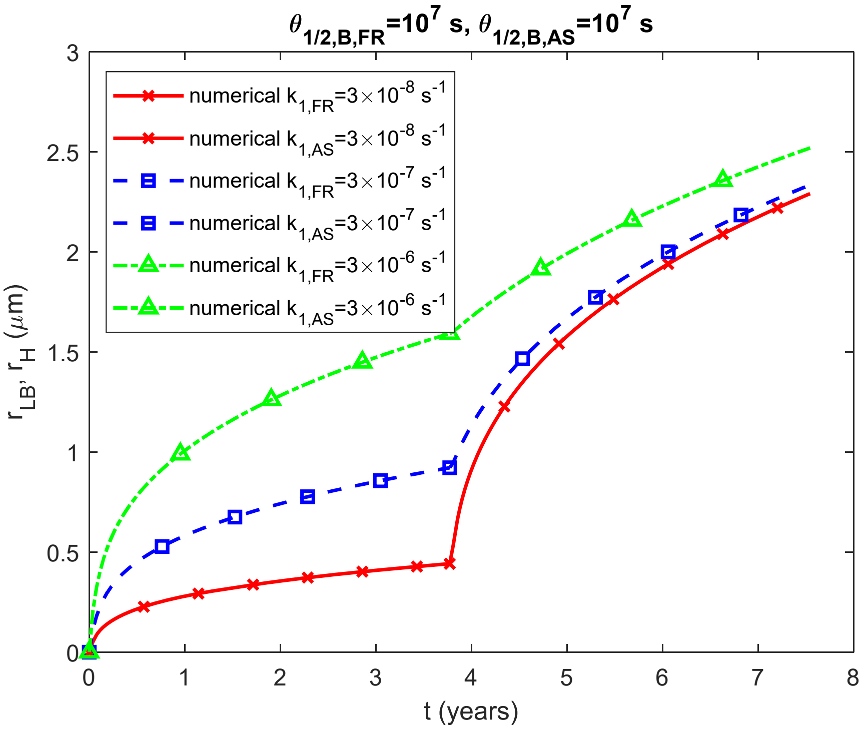

Fig. S2. (a) Molar concentrations of lipid membrane aggregates deposited into the core of the LB and α-syn aggregates deposited in the halo's fibrils, $[D_{FR}]$ and $[D_{AS}]$, respectively, for different values of $k_{1,FR}$ and $k_{1,AS}$ vs time. (b) Radii of the growing core of the LB and the growing halo, r_{LB} and r_H, respectively, for different values of $k_{1,FR}$ and $k_{1,AS}$ vs time. ($k_{2,FR} = k_{2,AS} = 2\times10^{-6}$ μM^{-1} s^{-1}, $q_{FR} = 1.57\times10^{-28}$ mol s^{-1}, $q_{AS} = 1.47\times10^{-21}$ mol s^{-1}.)

(a)

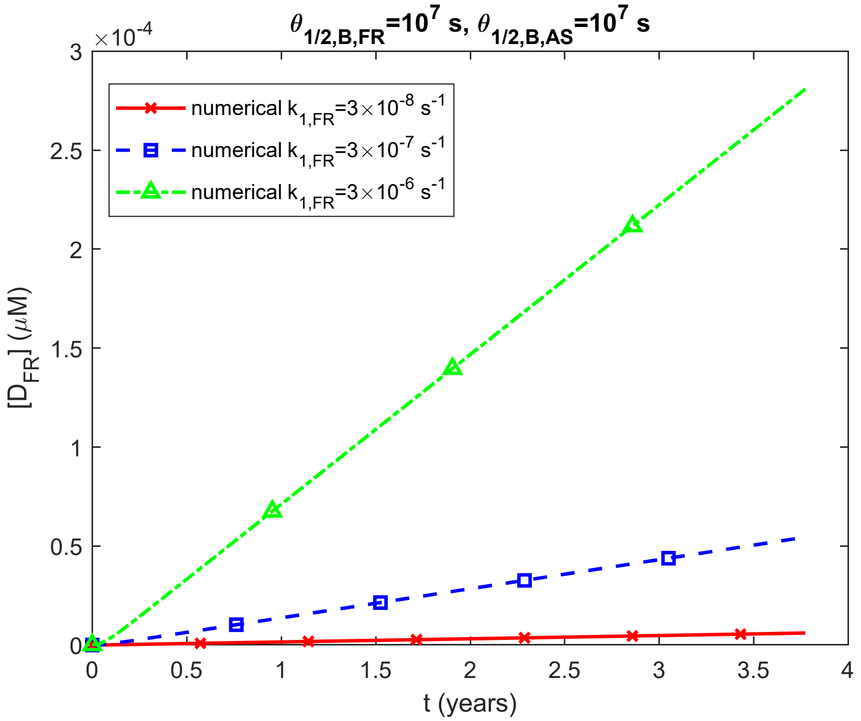

θ1/2,B,FR=10^7 s, θ1/2,B,AS=10^7 s
×10^-4
numerical k1,FR=3×10^-8 s^-1
numerical k1,FR=3×10^-7 s^-1
numerical k1,FR=3×10^-6 s^-1
[DFR] (μM)
t (years)

(b)

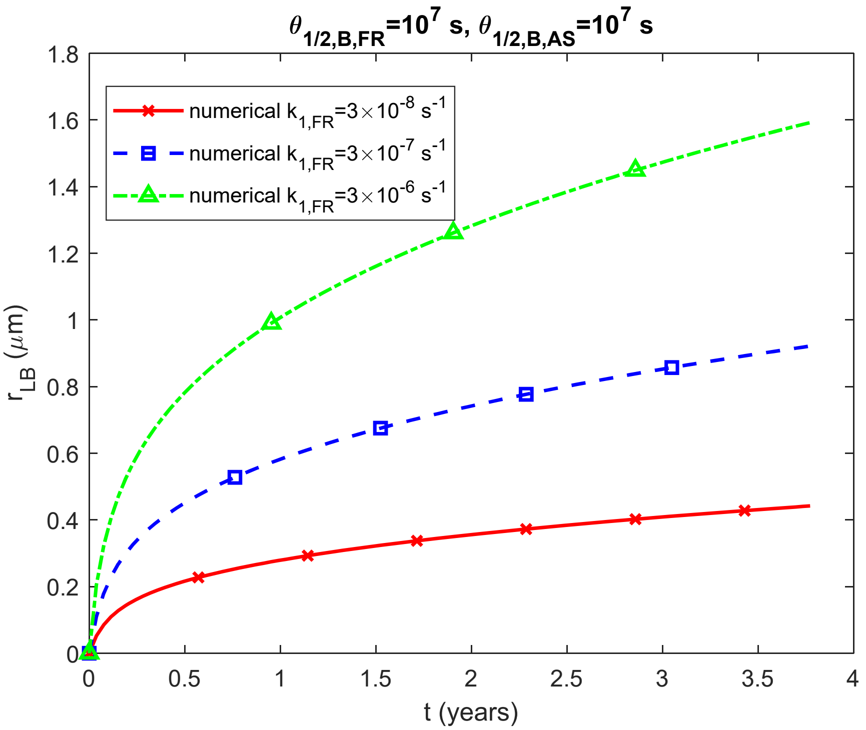

θ1/2,B,FR=10^7 s, θ1/2,B,AS=10^7 s
numerical k1,FR=3×10^-8 s^-1
numerical k1,FR=3×10^-7 s^-1
numerical k1,FR=3×10^-6 s^-1
rLB (μm)
t (years)

Fig. S3. (a) Molar concentration of lipid membrane aggregates deposited into the core of the LB, $[D_{FR}]$, for different values of $k_{1,FR}$ and $k_{1,AS}$ vs time. (b) Radius of the growing core of the LB, r_{LB}, for different values of $k_{1,FR}$ and $k_{1,AS}$ vs time. ($k_{2,FR} = k_{2,AS} = 2\times10^{-6}$ μM^{-1} s^{-1}, $q_{FR} = 1.57\times10^{-28}$ mol s^{-1}, $q_{AS} = 1.47\times10^{-21}$ mol s^{-1}.)

(a)

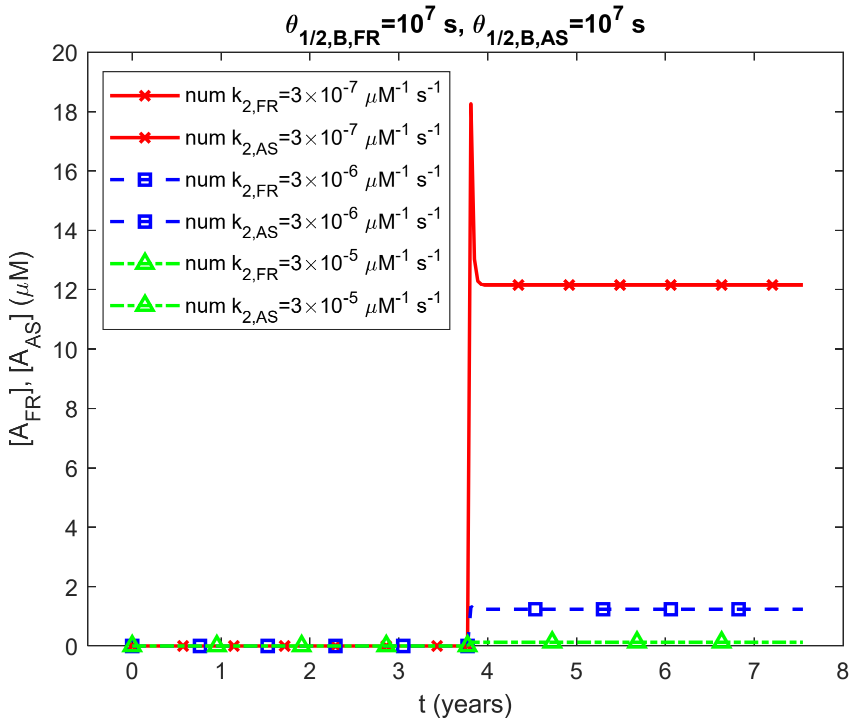

(b)

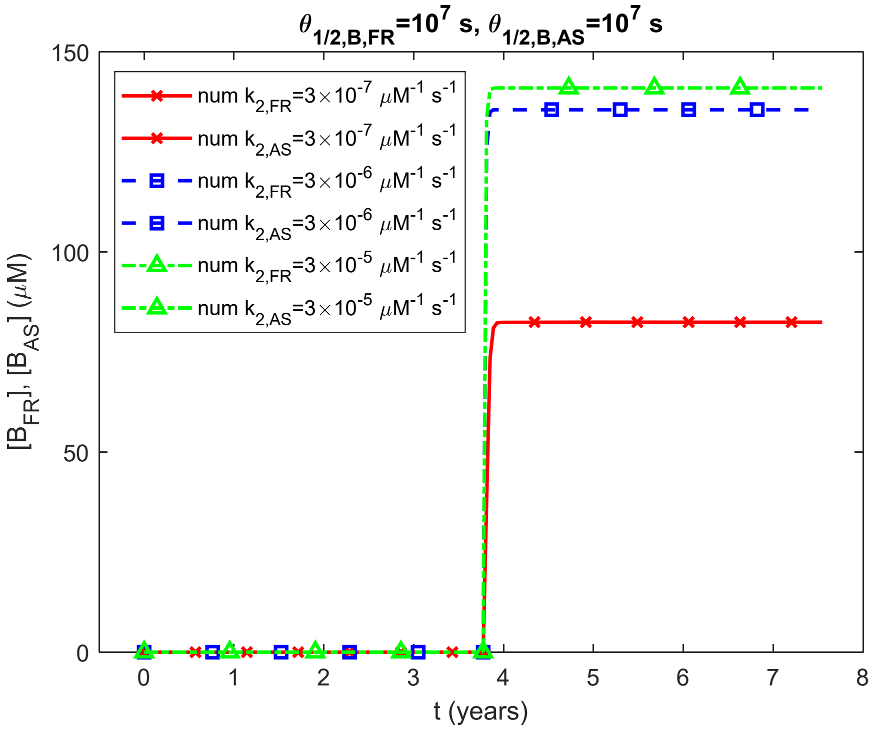

Fig. S4. (a) Molar concentrations of lipid membrane fragments and α-syn monomers, $[A_{FR}]$ and $[A_{AS}]$, respectively, for different values of $k_{2,FR}$ and $k_{2,AS}$ vs time. (b) Molar concentrations of free lipid membrane aggregates and free α-syn aggregates, $[B_{FR}]$ and $[B_{AS}]$, respectively, for different values of $k_{2,FR}$ and $k_{2,AS}$ vs time. ($k_{1,FR} = k_{1,AS} = 3\times10^{-7}$ s^{-1}, $q_{FR} = 1.57\times10^{-28}$ mol s^{-1}, $q_{AS} = 1.47\times10^{-21}$ mol s^{-1}.) In the legend, "num" stands for "numerical."

(a)

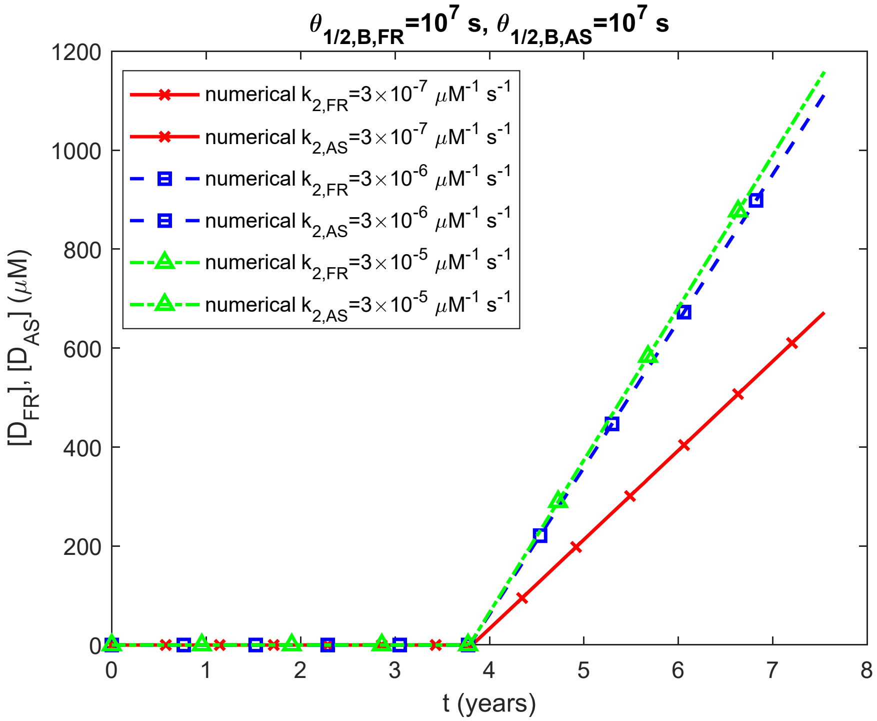

(b)

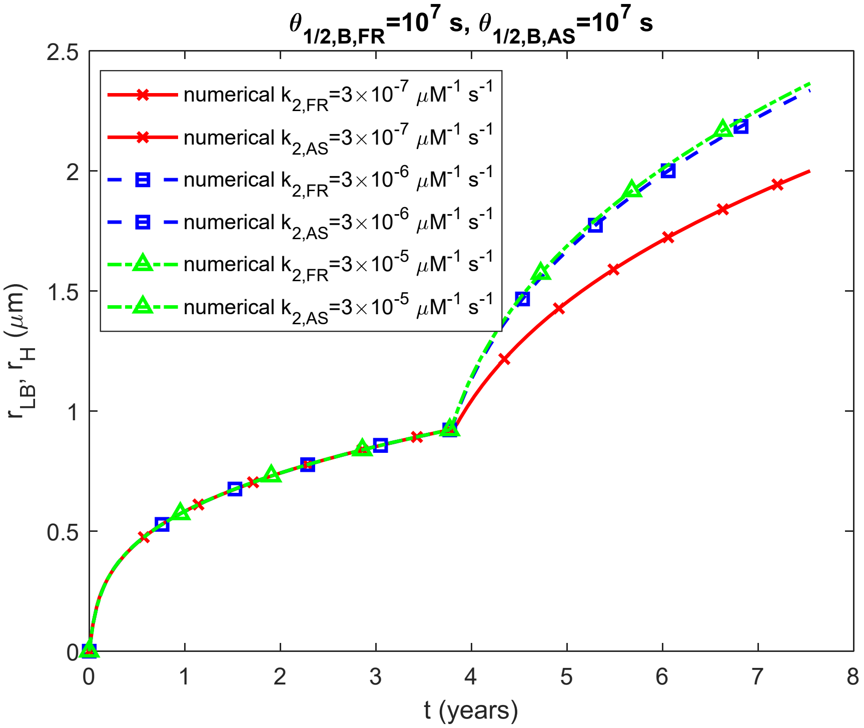

Fig. S5. (a) Molar concentrations of lipid membrane aggregates deposited into the core of the LB and α-syn aggregates deposited in the halo's fibrils, $\left[D_{FR}\right]$ and $\left[D_{AS}\right]$, respectively, for different values of $k_{2,FR}$

and $k_{2,AS}$ vs time. (b) Radii of the growing core of the LB and the growing halo, r_{LB} and r_H, respectively, for different values of $k_{2,FR}$ and $k_{2,AS}$ vs time. ($k_{1,FR} = k_{1,AS} = 3\times10^{-7}$ s^{-1}, $q_{FR} = 1.57\times10^{-28}$ mol s^{-1}, $q_{AS} = 1.47\times10^{-21}$ mol s^{-1}.)

(a)

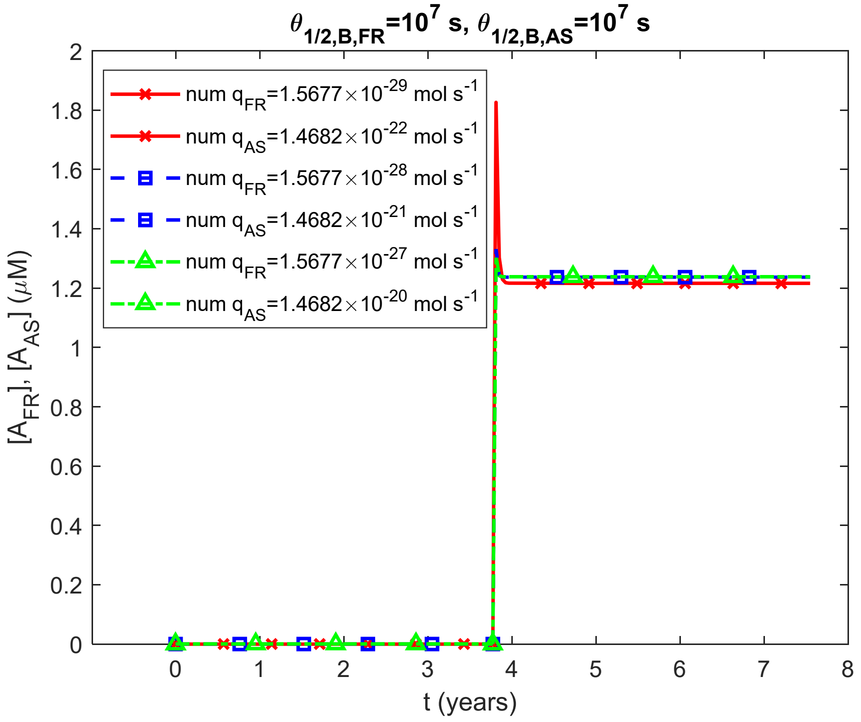

(b)

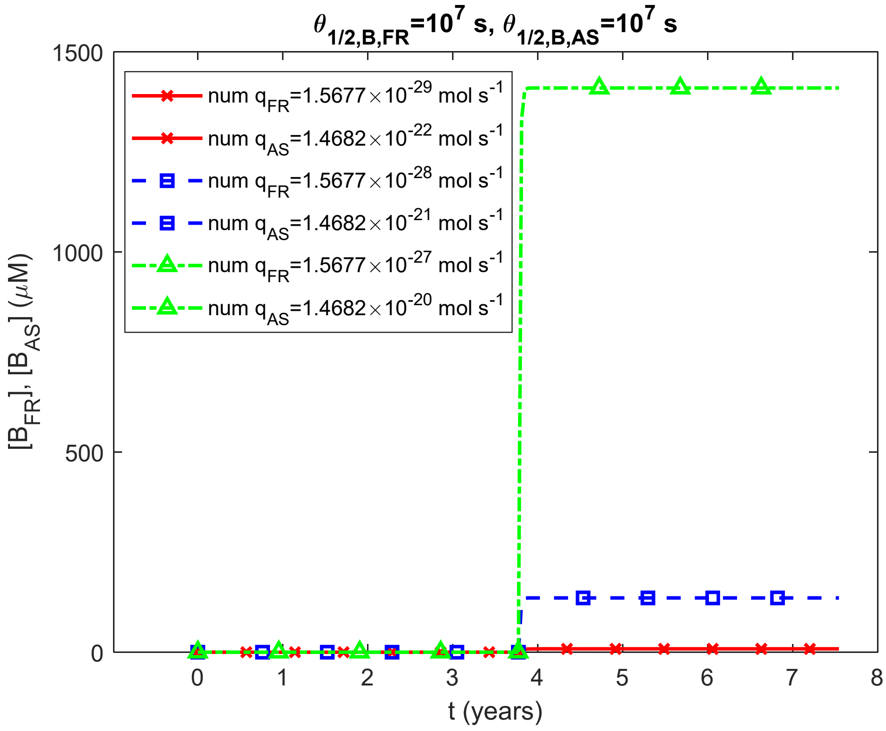

Fig. S6. (a) Molar concentrations of lipid membrane fragments and α-syn monomers, $[A_{FR}]$ and $[A_{AS}]$, respectively, for different values of q_{FR} and q_{AS} vs time. (b) Molar concentrations of free lipid membrane aggregates and free α-syn aggregates, $[B_{FR}]$ and $[B_{AS}]$, respectively, for different values of q_{FR} and q_{AS} vs time. ($k_{1,FR} = k_{1,AS} = 3\times10^{-7}$ s^{-1}, $k_{2,FR} = k_{2,AS} = 2\times10^{-6}$ μM^{-1} s^{-1}.) In the legend, "num" stands for "numerical."

(a)

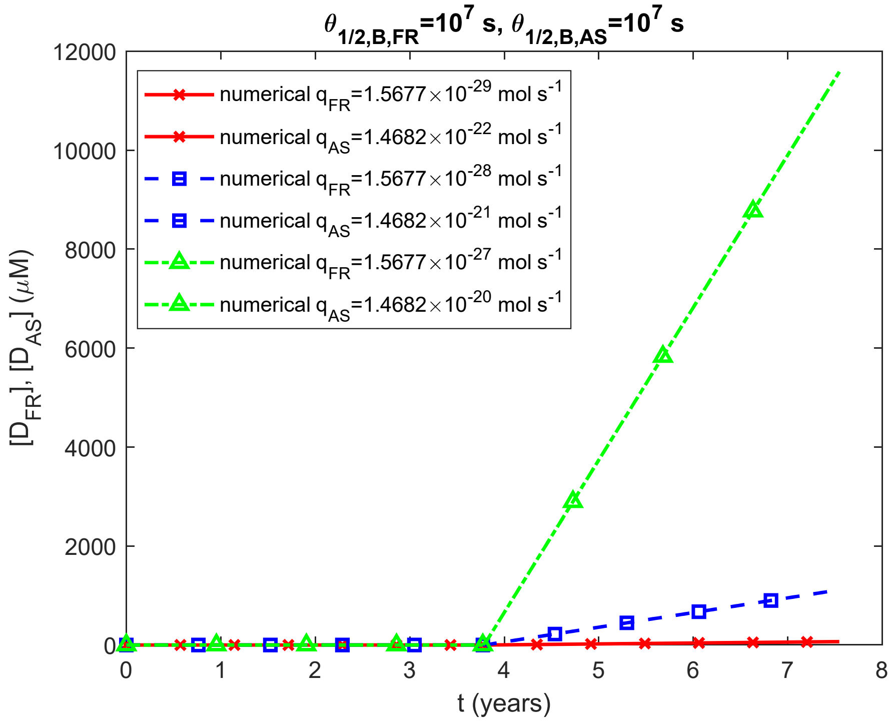

(b)

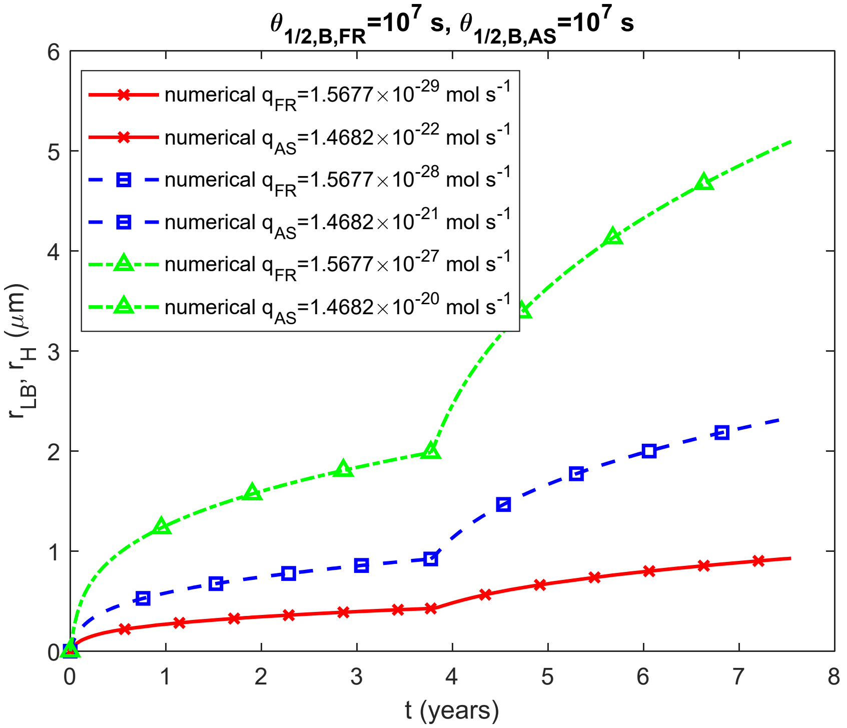

Fig. S7. (a) Molar concentrations of lipid membrane aggregates deposited into the core of the LB and α-syn aggregates deposited in the halo's fibrils, $\left[D_{FR}\right]$ and $\left[D_{AS}\right]$, respectively, for different values of q_{FR}

and q_{AS} vs time. (b) Radii of the growing core of the LB and the growing halo, r_{LB} and r_H, respectively, for different values of q_{FR} and q_{AS} vs time. ($k_{1,FR} = k_{1,AS} = 3\times10^{-7}$ s^{-1}, $k_{2,FR} = k_{2,AS} = 2\times10^{-6}$ μM^{-1} s^{-1}.)

(a)

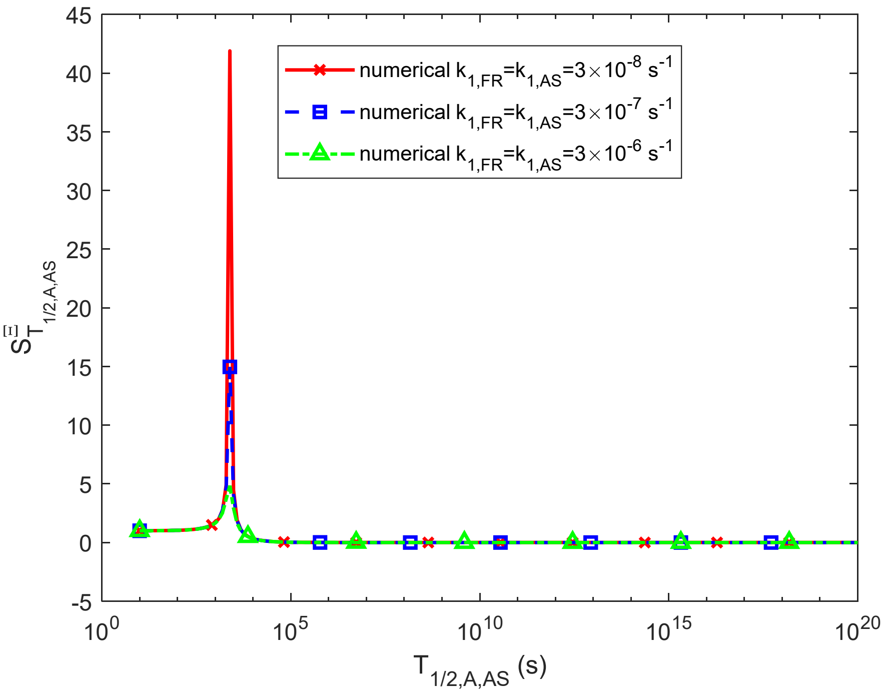

(b)

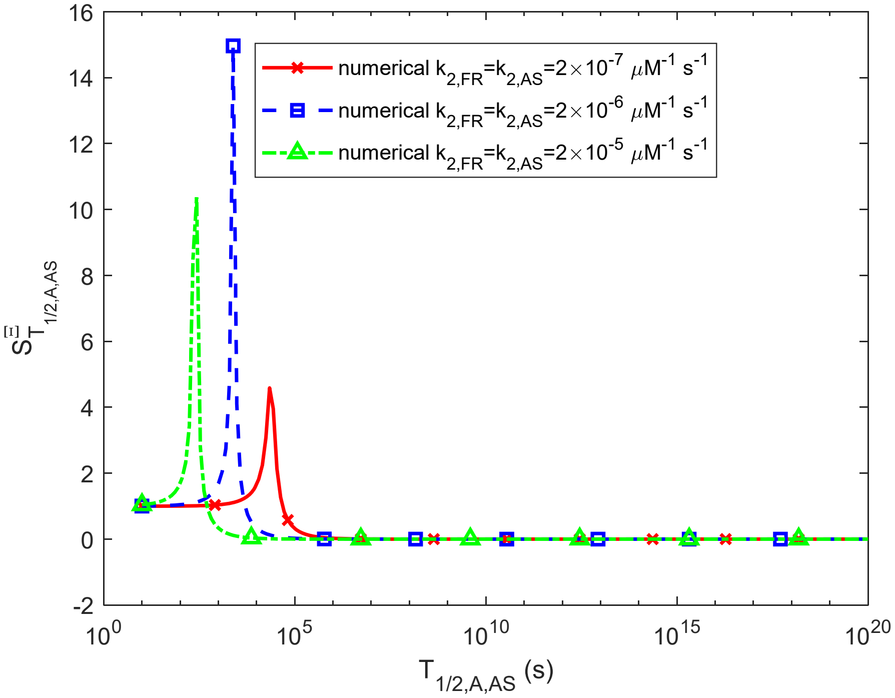

numerical k2,FR=k2,AS=2×10-7 μM-1 s-1
numerical k2,FR=k2,AS=2×10-6 μM-1 s-1
numerical k2,FR=k2,AS=2×10-5 μM-1 s-1
16
14
12
10
8
6
4
2
0
-2
10^0
10^5
10^10
10^15
10^20
T1/2,A,AS (s)

(c)

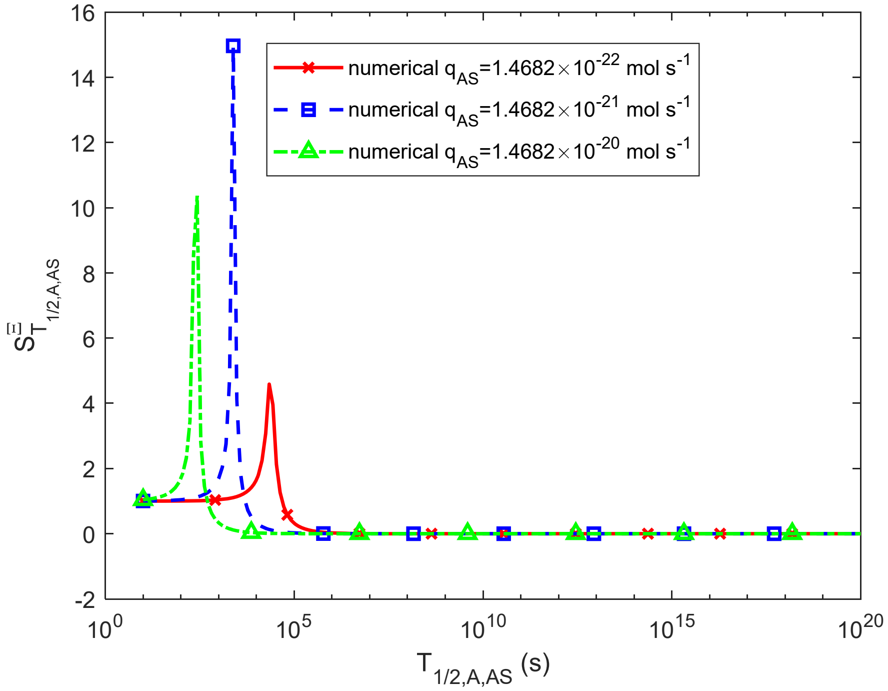

numerical qAS=1.4682×10-22 mol s-1
numerical qAS=1.4682×10-21 mol s-1
numerical qAS=1.4682×10-20 mol s-1
16
14
12
10
8
6
4
2
0
-2
10^0
10^5
10^10
10^15
10^20
T1/2,A,AS (s)

Fig. S8. Sensitivity of accumulated neurotoxicity, Ξ, to the half-life of α-syn monomers, $T_{1/2,A,AS}$, is examined for three different values of (a) the rate constants that describe the nucleation of membrane fragments and α-syn aggregates, $k_{1,FR}$ and $k_{1,AS}$ ($k_{2,FR}=k_{2,AS}=2\times10^{-6}$ μM^{-1} s^{-1}, $q_{FR}=1.57\times10^{-28}$ mol s^{-1}, $q_{AS}=1.47\times10^{-21}$ mol s^{-1}), (b) the rate constants that describe the autocatalytic growth of membrane fragments and α-syn aggregates, $k_{2,FR}$ and $k_{2,AS}$ ($k_{1,FR}=k_{1,AS}=3\times10^{-7}$ s^{-1}, $q_{FR}=1.57\times10^{-28}$ mol s^{-1}, $q_{AS}=1.47\times10^{-21}$ mol s^{-1}), (c) the production rates of membrane fragments and α-syn monomers, q_{FR} and q_{AS} . The following corresponding values were used for q_{FR}: $q_{FR}=1.57\times10^{-29}$ mol s^{-1} was used for $q_{AS}=1.47\times10^{-22}$ mol s^{-1}, $q_{FR}=1.57\times10^{-28}$ mol s^{-1} was used for $q_{AS}=1.47\times10^{-21}$ mol s^{-1}, and $q_{FR}=1.57\times10^{-27}$ mol s^{-1} was used for $q_{AS}=1.47\times10^{-20}$ mol s^{-1}. ($k_{1,FR}=k_{1,AS}=3\times10^{-7}$ s^{-1}, $k_{2,FR}=k_{2,AS}=2\times10^{-6}$ μM^{-1} s^{-1}). $T_{1/2,B,AS}$, $T_{1/2,A,FR}$, and $T_{1/2,B,FR}$ were kept at their values given in Table S2.

(a)

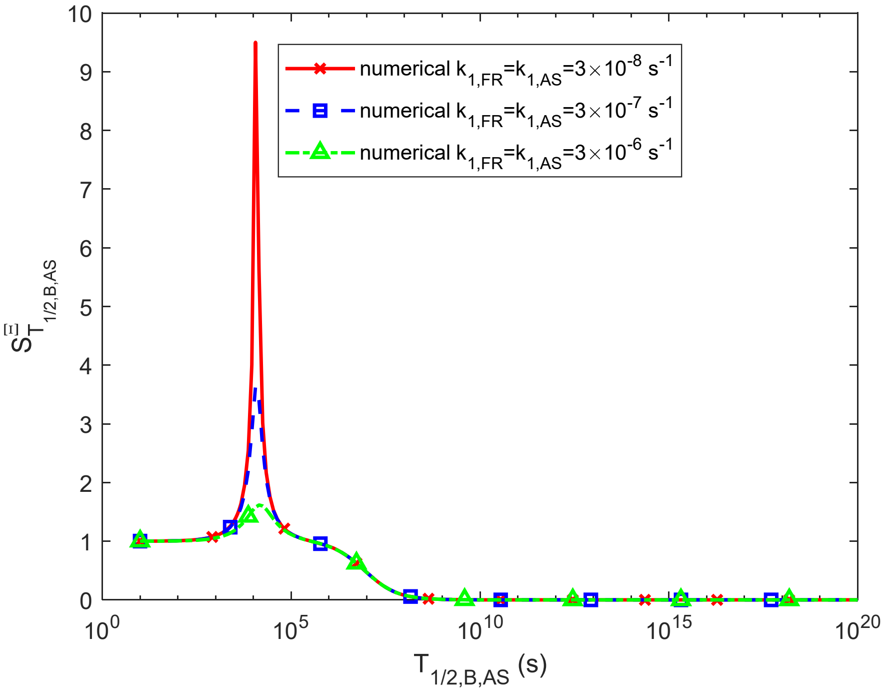

(b)

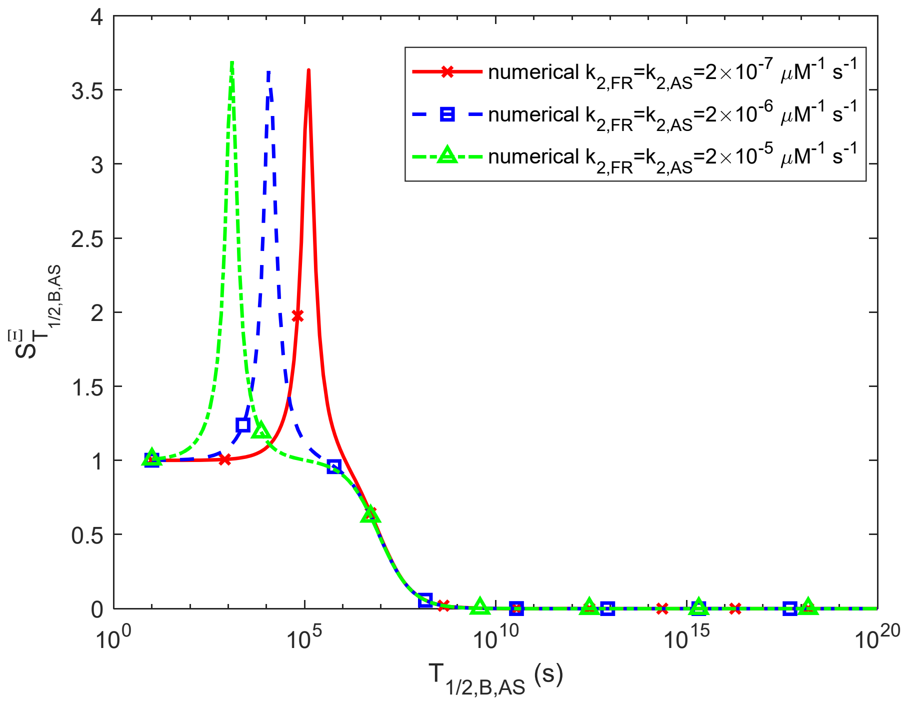

(c)

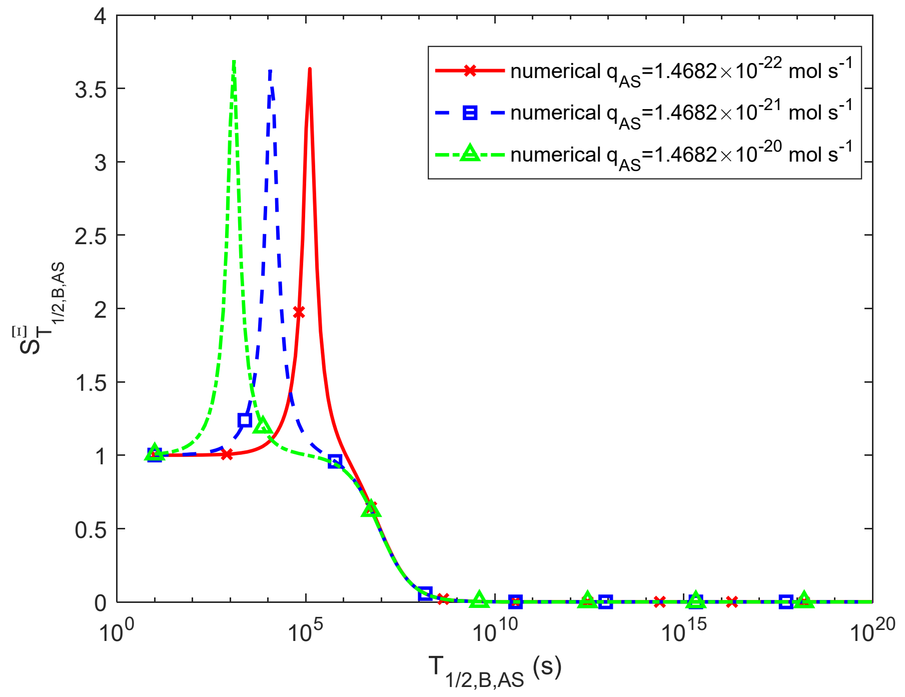

Fig. S9. Sensitivity of accumulated neurotoxicity, Ξ, to the half-life of free α-syn aggregates, $T_{1/2,B,AS}$, is examined for three different values of (a) the rate constants that describe the nucleation of membrane

fragments and α-syn aggregates, $k_{1,FR}$ and $k_{1,AS}$ ($k_{2,FR} = k_{2,AS} = 2\times10^{-6}$ μM-1 s-1, $q_{FR} = 1.57\times10^{-28}$ mol s-1, $q_{AS} = 1.47\times10^{-21}$ mol s-1), (b) the rate constants that describe the autocatalytic growth of membrane fragments and α-syn aggregates, $k_{2,FR}$ and $k_{2,AS}$ ($k_{1,FR} = k_{1,AS} = 3\times10^{-7}$ s-1, $q_{FR} = 1.57\times10^{-28}$ mol s-1, $q_{AS} = 1.47\times10^{-21}$ mol s-1), (c) the production rates of membrane fragments and α-syn monomers, q_{FR} and q_{AS}. The following corresponding values were used for q_{FR}: $q_{FR} = 1.57\times10^{-29}$ mol s-1 was used for $q_{AS} = 1.47\times10^{-22}$ mol s-1, $q_{FR} = 1.57\times10^{-28}$ mol s-1 was used for $q_{AS} = 1.47\times10^{-21}$ mol s-1, and $q_{FR} = 1.57\times10^{-27}$ mol s-1 was used for $q_{AS} = 1.47\times10^{-20}$ mol s-1. ($k_{1,FR} = k_{1,AS} = 3\times10^{-7}$ s-1, $k_{2,FR} = k_{2,AS} = 2\times10^{-6}$ μM-1 s-1.) $T_{1/2,A,AS}$, $T_{1/2,A,FR}$, and $T_{1/2,B,FR}$ were kept at their values given in Table S2.
